# Microcavity phonon polaritons from the weak to the ultrastrong phonon–photon coupling regime

**DOI:** 10.1038/s41467-021-26060-x

**Published:** 2021-10-27

**Authors:** María Barra-Burillo, Unai Muniain, Sara Catalano, Marta Autore, Fèlix Casanova, Luis E. Hueso, Javier Aizpurua, Ruben Esteban, Rainer Hillenbrand

**Affiliations:** 1 CIC nanoGUNE BRTA, 20018 Donostia - San Sebastián, Spain; 2 Donostia International Physics Center, 20018 Donostia-San Sebastián, Spain; 3 IKERBASQUE, Basque Foundation for Science, 45011 Bilbao, Spain; 4 Materials Physics Center, CSIC-UPV/EHU, 20018 Donostia-San Sebastián, Spain; 5 CIC nanoGUNE BRTA and Department of Electricity and Electronics, EHU/UPV, 20018 Donostia-San Sebastián, Spain

**Keywords:** Nanoscale materials, Microresonators, Polaritons

## Abstract

Strong coupling between molecular vibrations and microcavity modes has been demonstrated to modify physical and chemical properties of the molecular material. Here, we study the less explored coupling between lattice vibrations (phonons) and microcavity modes. Embedding thin layers of hexagonal boron nitride (hBN) into classical microcavities, we demonstrate the evolution from weak to ultrastrong phonon-photon coupling when the hBN thickness is increased from a few nanometers to a fully filled cavity. Remarkably, strong coupling is achieved for hBN layers as thin as 10 nm. Further, the ultrastrong coupling in fully filled cavities yields a polariton dispersion matching that of phonon polaritons in bulk hBN, highlighting that the maximum light-matter coupling in microcavities is limited to the coupling strength between photons and the bulk material. Tunable cavity phonon polaritons could become a versatile platform for studying how the coupling strength between photons and phonons may modify the properties of polar crystals.

## Abstract (web summary)

Strong coupling between light and matter can be engineered to influence their properties and behaviour. Here, the authors demonstrate the evolution from weak to ultrastrong coupling of microcavity modes and optical phonons with hexagonal boron nitride layers in a Fabry-Perot resonator.

## Introduction

When light strongly couples to matter, new hybrid modes – polaritons - can emerge, whose coherent exchange of energy is faster than the decay rate of the original photonic modes and matter excitations^1,2^. Typically, polariton waves are created by placing matter inside an optical resonator, such as a Fabry–Perot microcavity^3,4^. The interaction between visible light and excitonic systems has been widely explored over the past decades^5,6^, leading to fundamental and applied advancements in the field, including the realization of polariton Bose–Einstein condensates^7,8^ and various excitonic LASER systems^9^. In analogue fashion, strong coupling in the infrared regime has been explored in semiconductor quantum wells^10–13^, polar dielectrics^14^, and molecular aggregates^15^. In particular, strong coupling (SC) between infrared light and molecular vibrations (vibrational strong coupling, VSC) has emerged as a new intriguing research topic, after it has been reported that this phenomenon can lead to modification of fundamental material properties, triggering, for example, phase transitions^16^ or modifying chemical reactions^17,18^. In the reported experiments, strong coupling was achieved by filling classical Fabry–Pérot microcavities with molecules. Recently, the molecular vibrational strong coupling could be achieved even on the nanometre scale, by exploiting phonon polaritons in hexagonal boron nitride (hBN) nanoresonators^19^ and slabs^20^, the phonon polaritons by themselves being the results of strong coupling between infrared photons and phonons.

The strong and ultrastrong coupling between light and phonons offers intriguing possibilities for various fundamental studies and applications, including polaritonic control of THz waves in polar crystals^21^, which in combination with microresonators^22,23^, can be used for the development of phonon polariton lasers^24,25^. At surfaces or on thin layers of polar crystals, strong phonon–photon coupling can also lead to surface phonon polaritons and hyperbolic volume phonon polaritons^26,27^ that allow for nanoscale concentration of infrared and terahertz fields, which could lead to novel communication and sensing technologies^28,29^, particularly in form of nanoresonators^26,30,31^ or by coupling the polaritons with plasmonic antennas and metasurfaces^32–36^. Remarkably, a detailed study and control of the coupling strength between photons and phonons in classical Fabry–Pérot microcavities is relatively unexplored terrain. This might be related with the difficulty to fabricate high-quality thin crystal layers of arbitrary thickness and place them inside the microcavities. As phonons have significant influence on many physical properties of crystals^37^, such as on electrical and thermal conductivity, ferroelectricity^38,39^, and superconductivity^40^, controlling the coupling strength between infrared photons and phonons in microcavities may become an interesting platform for future fundamental and applied studies. For example, recent studies suggest that cavity-mediated strong light-phonon coupling may trigger quantum phase transitions without the need of an external pump^16,41^.

Here we demonstrate infrared microcavities comprising polar van der Waals (vdW) materials as a versatile test bench to study the interaction of optical phonons and photons. Importantly, the layered structure of vdW materials allows for convenient exfoliation of high-quality crystalline layers of virtually any thickness for studying the evolution of the phonon–photon coupling strengths as a function of layer thickness. Specifically, we study in this work microcavities containing high-quality layers of hBN, which is an insulating polar material exhibiting phonons in the mid-infrared (IR) spectral range^42^. The experimental reflectivity of the hBN-microcavity system is well described by electrodynamical calculations based on the transfer matrix method^43^. Furthermore, by describing this system by two classical coupled harmonic oscillators^44–46^, we estimate the coupling strength between the cavity modes and the phonon excitation. We demonstrate that strong coupling can be achieved for layers as thin as a few nanometres, leading to the formation of microcavity phonon polaritons. We further systematically trace the evolution from the weak to the ultrastrong coupling (USC) regime^47–50^. The high crystal quality and adjustable layer thickness, both achieved by simple exfoliation, establish microcavities embedding van der Waals materials as a versatile platform for studying and tuning the coupling strengths between photons and optical phonons.

## Results

### Infrared spectroscopy and analysis of hBN filled microcavities

**Fig. 1:**
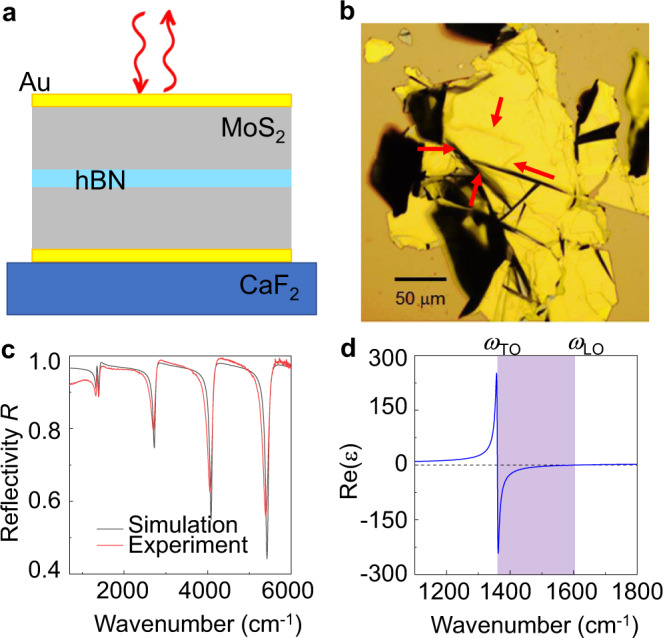
Cavity phonon polariton experiment. **a** Sketch of the experiment, illustrating spectroscopy of the infrared light reflected at a microcavity made of two gold mirrors embedding an hBN slab. **b** Optical light microscope image of a representative sample. The arrows mark the boundaries of the microcavity where the hBN flake is embedded in between two MoS_2_ flakes. **c** Experimental (red) and simulated (black) infrared reflection spectrum of a cavity embedding a 10 nm thick hBN layer. **d** Real part of the in-plane dielectric function of hBN, *ε*, taken from ref. ^19^. The purple area highlights the Reststrahlen band (RB) between the transverse optical (TO) and longitudinal optical (LO) phonon frequencies.

We illustrate the microcavities and the infrared reflection spectroscopy measurements in Fig. 1. The schematic in Fig. 1a represents a thin hBN flake sandwiched between two molybdenum disulfide (MoS_2_) layers, resulting in a MoS_2_/hBN/MoS_2_ dielectric stack, with the hBN flake being located in the middle. Such heterostructures are fabricated following several steps of mechanical exfoliation on polydimethylsiloxane (PDMS) and deterministic transfer^51^. We use MoS_2_ as a spacer, as it is spectrally flat in the mid-IR spectral region and can be easily obtained by exfoliation. An optical cavity is formed by placing the MoS_2_/hBN/MoS_2_ dielectric stack in between two gold layers of 20 nm thickness, fabricated by thermal evaporation. To locate the hBN flake in the cavity center—where the maximum intensity of the electric field is expected to occur for odd cavity modes—we chose MoS_2_ flakes of ideally the same thickness. Details of the fabrication process can be found in the Methods section. For the present study, we fabricated cavities embedding hBN flakes of varying thickness ${L}_{{{{{{\rm{hBN}}}}}}}$. For each cavity, the total cavity length$\,{L}_{{{{{{\rm{cav}}}}}}}$ was adjusted such that the fundamental cavity resonance ${\omega }_{{{{{{\rm{cav}}}}}}}^{\left(1\right)}$ coincides with the frequency of the in-plane transverse optical (TO) phonon of hBN, ${\omega }_{{{{{{\rm{TO}}}}}}}$ = 1,364 cm^–^^1^ (169.1 meV) (Fig. 1d)^26,42^. We determined ${L}_{{{{{{\rm{cav}}}}}}}$ by calculating the modes of a virtual cavity (in the following referred as to a bare cavity), for which we assume a frequency-independent hBN permittivity, ${\varepsilon \left(\omega \right)=\varepsilon }_{{{{{{\rm{hBN}}}}}},\infty}=$ 4.52 that neglects the phonon contribution (see “Supplementary Note 1” for details).

Figure 1b shows an optical microscope image (top view) of a representative sample, where the arrows mark the microcavity containing the hBN layer. With a lateral size of typically more than 25 μm, it is large enough to perform reliable Fourier transform infrared (FTIR) microspectroscopy (illustrated in Fig. 1a). In our experiments, we recorded normal-incidence reflection spectra with an FTIR setup (Bruker Hyperion 2000 infrared microscope coupled to a Bruker Vertex 70 FTIR spectrometer) that operates with a Cassegrainian objective with the numerical aperture of NA = 0.4 (not shown in Fig. 1a). An example spectrum (red curve) is shown in Fig. 1c, which was obtained from a cavity embedding a 10 nm hBN layer between 510 nm and 370 nm thick MoS_2_ layers. For frequencies well above the TO phonon frequency, we see a clear series of reflectivity dips that can be well matched by electrodynamical calculations employing the Transfer Matrix (TM) method (black spectrum in Fig. 1c) at normal incidence. Each of the dips can be attributed to a specific cavity mode (Supplementary Note 2). Interestingly, at the TO phonon frequency, both experimental and simulated spectra reveal a double-dip feature, indicating that coupling between the hBN TO phonon and the fundamental cavity mode can be achieved with our heterostructures.

**Fig. 2:**
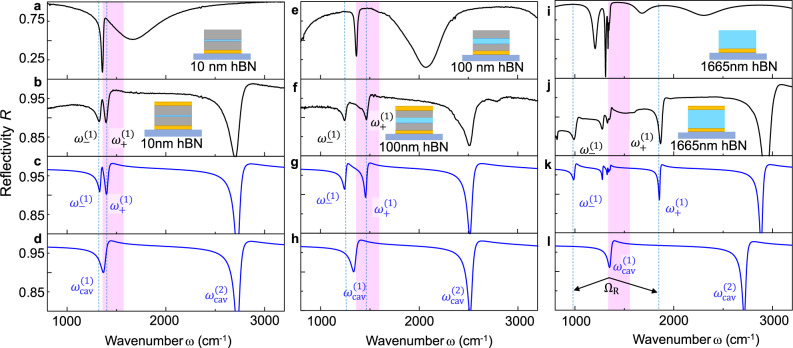
Zoom into experimental and calculated spectra at frequencies close to the TO phonon. **a** Experimental reflectivity spectrum of the MoS_2_/hBN/MoS_2_ heterostructure placed on the bottom gold mirror (illustrated by inset), measured before the evaporation of the top gold mirror. Measured thicknesses are 510 nm/10 nm/370 nm. **b** Experimental reflectivity spectrum of the stack of the panel (**a**) after closing it with the top Au layer (illustrated by inset). ${\omega }_{-}^{(1)}$ and ${\omega }_{+}^{(1)}$ mark the dips emerging for the coupling between TO phonon and cavity mode. **c** Simulated reflectivity spectrum of the cavity in panel (**b**), using layer thicknesses of 510 nm/10 nm/370 nm. **d** Simulated reflection spectrum of the cavity of the panel (**c**), in which hBN was replaced by a dielectric medium with ${\varepsilon \left(\omega \right)=\varepsilon }_{{{{{{\rm{hBN}}}}}},{{\infty }}}=$ 4.52. The labels ${\omega }_{{{{{{\rm{cav}}}}}}}^{(1)}$ and ${\omega }_{{{{{{\rm{cav}}}}}}}^{(2)}$ mark the first and second-order cavity mode. **e**–**h** Same as panels (**a**–**d**) for a MoS_2_/hBN/MoS_2_ heterostructure with measured thicknesses 520 nm/100 nm/430 nm and simulated thicknesses 480 nm/100 nm/390 nm. **i**–**l** Same as panels (**a**–**d**) for a cavity fully filled with hBN, with measured and simulated hBN thickness 1,665 nm. ${\Omega }_{R}={\omega }_{+}^{(1)}-{\omega }_{-}^{(1)}$ in panel **l** marks the dip splitting. Black and blue curves show experimental and simulation results, respectively, and purple shaded areas highlight the Reststrahlen band.

To explore in further detail the coupling between the fundamental cavity mode and the hBN phonon, we performed a combined experimental and numerical study of cavities embedding 10 nm, 100 nm, and 1,665 nm thick hBN flakes (for sketches see insets in Fig. 2b, f, j). We first verify that all three hBN flakes exhibit a sharp phonon line at ${\omega }_{{{{{{\rm{TO}}}}}}}$ = 1,364 cm^–^^1^, by measuring reflection spectra of the stacks prior to the fabrication of the top mirror (Fig. 2a, e, i). Additional to the phonon lines, we observe broader dips, which indicate that the stacks act as detuned open cavities of a low quality factor. By closing the cavities (i.e. fabricating the top gold mirror), we clearly see a splitting of the reflection dip at the TO phonon frequency into two dips that are shifted to a lower and a higher frequency, ${\omega }_{-}^{(1)}\,{{{{{\rm{and}}}}}}\,{\omega }_{+}^{(1)}$, respectively (vertical blue dashed lines in Fig. 2b, f, j). Transfer matrix calculations match well the experimental reflection spectra (Fig. 2c, g, k) upon slight modification of the nominal values of the cavity parameters (see caption of Fig. 2). The need for such modification is attributed to uncertainties in the thickness and permittivity measurements. We find that the dip splitting ${\omega }_{+}^{(1)}-{\omega }_{-}^{(1)}$ significantly increases with increasing hBN thickness, in the experiment from 76 cm^–^^1^ to 215 cm^–^^1^ to 864 cm^–^^1^ (Fig. 2b, f, j) and in the calculations from 68 cm^–^^1^ to 213  cm^–^^1^ to 860 cm^–^^1^ (Fig. 2c, g, k). For comparison, we show in Fig. 2d, h, l transfer matrix calculations of the bare cavity reflectivity, revealing that the uncoupled cavity modes indeed nearly perfectly coincide with the TO phonon frequency of the hBN layers, demonstrating that the double-dip feature is a consequence of coupling between TO phonon and the respective fundamental cavity resonance. In the cavity that is fully filled with hBN (Fig. 2j, k), the TO phonon also couples with higher-order cavity modes, which leads to the appearance of additional dips close to the TO phonon frequency (see discussion of Fig. 5).

**Fig. 3:**
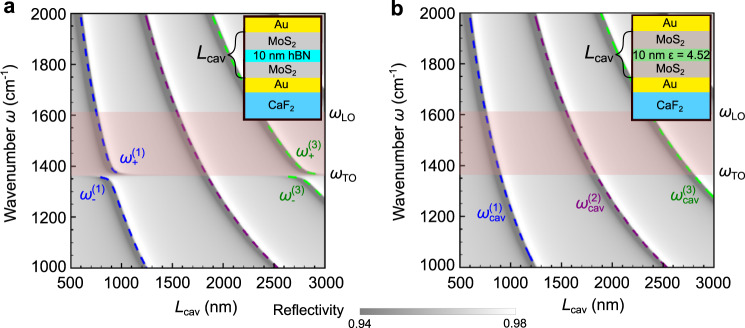
Strong coupling in a cavity filled with 10 nm of hBN. **a** TM simulated reflectivity spectra of a cavity with a 10 nm thick hBN layer in its center (illustrated by inset) as a function of the total cavity thickness ${L}_{{{{{{\rm{cav}}}}}}}$. The dashed curves show the cavities’ eigenmode frequencies $\,{\omega }_{+}^{(j)}$ and ${\omega }_{-}^{(j)}$ as a function of ${L}_{{{{{{\rm{cav}}}}}}}$. The reddish areas in (**a**) and (**b**) mark the Reststrahlen band of the hBN in-plane phonon. **b** TM simulated reflectivity spectra of a bare cavity (illustrated by inset) as a function of cavity thickness ${L}_{{{{{{\rm{cav}}}}}}}$. The dashed curves show the cavities’ eigenmode frequencies $\,{\omega }_{{{{{{\rm{cav}}}}}}}^{(j)}$ as a function of ${L}_{{{{{{\rm{cav}}}}}}}$.

**Fig. 4:**
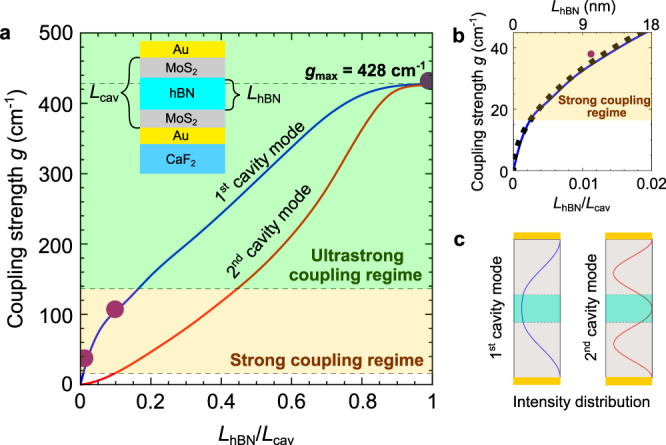
Evolution of the coupling strength as a function of the cavity filling factor. **a** Coupling strength $g$ between the TO phonon and the bare-cavity modes, for an hBN layer of thickness ${L}_{{{{{{\rm{hBN}}}}}}}$ in the middle of a cavity of thickness ${L}_{{{{{{\rm{cav}}}}}}}$, as sketched in the inset. The calculated evolution of $g$ for varying ${L}_{{{{{{\rm{hBN}}}}}}}$ is shown for the first (blue solid line) and the second (red solid line) cavity modes. The coupling strengths corresponding to the strong coupling regime are highlighted by the beige area, and the green area corresponds to the ultrastrong coupling regime. Purple symbols indicate the experimental coupling strength ${g}_{{{\exp }}}$ obtained from Fig. 2. **b** Zoom into the panel (**a**) for small filling factors ${L}_{{{{{{\rm{hBN}}}}}}}/{L}_{{{{{{\rm{cav}}}}}}}$, showing the calculated (blue line) and experimental (purple symbol) coupling strength $g\,$for the first cavity mode. The analytical approximation $g\approx 332\sqrt{{L}_{{{{{{\rm{hBN}}}}}}}/{L}_{{{{{{\rm{cav}}}}}}}}$ cm^−^^1^ is shown by black dots. **c** Sketch of the intensity distribution of the electric field of the first and second cavity modes. We note that the hBN layer is placed at the maximum of the intensity of the first cavity mode and at the minimum for the second cavity mode.

We further corroborate the coupling between the TO phonon and the cavity modes by showing in Fig. 3 the evolution of the reflectivity spectra as the cavity resonance is detuned from the TO phonon. For this purpose, we performed TM simulations of the reflectivity spectra as a function of total cavity length, ${L}_{{{{{{\rm{cav}}}}}}}$, while keeping fixed the thickness of the hBN layer that is placed in the middle of the cavity, ${L}_{{{{{{\rm{hBN}}}}}}}$ $=$ 10 nm (Fig. 3a, contour plot). We performed similar calculations for the bare cavity (Fig. 3b, contour plot) and compare the results. One can observe that the first ($j$  = 1) and third ($j$ = 3) bare cavity modes (${\omega }_{{{{{{\rm{cav}}}}}}}^{\left(j\right)}$ in Fig. 3b) split into an upper and a lower branch when the hBN layer is included (${\omega }_{-}^{\left(j\right)}$ and ${\omega }_{+}^{\left(j\right)}$ in Fig. 3a), as recognized by the reflection dips and their corresponding resonance frequencies (colored dashed lines in Fig. 3a, b). Most important, the anti-crossing of the branches at the TO phonon frequency manifests the typical signature of coupling between two modes. The second cavity mode, in contrast, does not show any splitting of the reflection dips and thus reveals the absence of coupling, which can be explained by the vanishing electric field intensity of this mode in the cavity center (for further details see discussion of Fig. 4c). The same analysis and analogue observations were made for the cavity embedding a 100 nm thick hBN layer (Supplementary Note 3). Furthermore, we study the dependence of the reflectivity spectra on the incident angle in Supplementary Note 5.

### Evolution of coupling strength with cavity filling factor

For a quantitative analysis of the coupling between the hBN phonons and the microcavity photons in the TM calculations, we modeled the phonon–photon interaction by two coupled harmonic oscillators. One oscillator is associated with the electromagnetic field of the cavity (characterized by its resonance frequency ${\omega }_{{{{{{\rm{cav}}}}}}}^{\left(j\right)}$ and decay rate $\kappa =$ 60 cm^–^^1^) and the other one with the hBN phonon (characterized by its resonance frequency ${\omega }_{{{{{{\rm{TO}}}}}}}\,$ and decay rate $\gamma =$ 5 cm^–^^1^). This model yields the eigenfrequencies ${\omega }_{+}^{\left(j\right)}$ and ${\omega }_{-}^{\left(j\right)}$ as a function of the known parameters ${\omega }_{{{{{{\rm{cav}}}}}}}^{\left(j\right)}$, ${\omega }_{{{{{{\rm{TO}}}}}}},\kappa$, $\gamma$ and the unknown coupling strength $g$. Using ${\omega }_{+}^{\left(j\right)}$ and ${\omega }_{-}^{\left(j\right)}$ obtained from the TM calculations, we can apply the coupled harmonic oscillator model for determining $g$ (see “Methods” section and “Supplementary Note 2.3”).

We first studied the evolution of the coupling strength with the hBN layer thickness ${L}_{{{{{{\rm{hBN}}}}}}}$. To that end, we calculated $g$ as a function of the filling factor ${f={L}}_{{{{{{\rm{hBN}}}}}}}/{L}_{{{{{{\rm{cav}}}}}}}$ for the first and second cavity modes (blue and red solid lines in Fig. 4a, respectively). We observe that $g$ increases with the filling factor for both modes, more strongly for the first mode, which is a consequence of the intensity distribution of the electric field across the cavity (Fig. 4c). The second cavity mode exhibits an intensity minimum in the center of the cavity, where the hBN layer is located. Thus, the coupling between hBN phonons and second cavity mode is generally much smaller than for the first cavity mode, that exhibits its intensity maximum in the cavity center. Note that this observation is consistent with the absence of a polariton gap for the second cavity mode in Fig. 3a. The interaction between the TO phonon and the second cavity mode remains weak until the hBN layer is thick enough to sufficiently overlap with the cavities’ off-center intensity maxima (shown in right panel of Fig. 4c). A larger coupling strength can also be achieved for the second cavity mode by distributing the hBN layer near the positions where the intensity of this mode is maximum^52^ (we discuss the dependence of the coupling strength on the layer position in Supplementary Note 7). Interestingly, the coupling strength $g$ for both the first and second cavity mode can be well approximated by an analytical expression obtained by a microscopic theory that is described in Supplementary Note 6.

We can identify the weak and strong coupling regimes in Fig. 4a according to the fulfillment of the conditions $\frac{g}{\kappa +\gamma } \, < \, \frac{1}{4}$ (blank area) and $\frac{g}{\kappa +\gamma } \, > \, \frac{1}{4}$ (beige area), respectively^1,5,53^. For the first cavity mode, remarkably, the strong coupling regime starts for ${f=L}_{{{{{{\rm{hBN}}}}}}}/{L}_{{{{{{\rm{cav}}}}}}}$ ≈ 0.0025, which corresponds to hBN slabs of about 3 nm thickness (about four atomic hBN layers). Moreover, the usual condition for ultrastrong coupling, $g$/${\omega }_{{{{{{\rm{TO}}}}}}}$ > 0.1^47,54^ (highlighted by the green area in Fig. 4), is fulfilled for hBN layers of thickness ${L}_{{{{{{\rm{hBN}}}}}}}\,$ > 148 nm. Interestingly, we find that $g$ saturates for the first-order mode when$\,f$ > 0.8 and for the second-order mode when $f$ > 0.9. The maximum coupling strength is obtained for both modes when the cavity is fully filled with hBN, with ${g}_{{{\max }}}=\sqrt{\frac{{\omega }_{{{{{{\rm{LO}}}}}}}^{2}-{\omega }_{{{{{{\rm{TO}}}}}}}^{2}\,}{4}}=$ 428 cm^–^^1^ being exclusively determined by the materials’ Reststrahlen band (RB) defined by the TO and LO phonon frequencies (for further discussion see below). In this case, the ratio ${g}_{{{\max }}}$/${\omega }_{{{{{{\rm{TO}}}}}}}$ reaches up to 0.31. These results show the versatility of the hBN-filled microcavities to explore the different light-matter coupling regimes, ranging from weak to ultrastrong coupling.

Figure 4b shows a zoom into Fig. 4a for small filling factors (when the intensity distribution can be assumed homogeneous within the hBN), where we observe that the evolution of the coupling strength of the first cavity mode scales with $\sqrt{{L}_{{{{{{\rm{hBN}}}}}}}}\,$ (solid dots) for small thicknesses. This behavior is consistent with the well-known scaling law of strong coupling^55–57^, $g \sim \sqrt{N}$, where $N$ is the number of oscillators, which in the case of an hBN slab is proportional to ${L}_{{{{{{\rm{hBN}}}}}}}$ (see “Supplementary Note 6.2” for the derivation of a simplified analytical expression for very small and very large filling factors).

To identify the coupling regime for the experimental spectra shown in Fig. 2, we determine the coupling strength according to ${g}_{{{{{{\rm{exp }}}}}}}=({\omega }_{+}^{(1)}-{\omega }_{-}^{(1)})/2$ where ${\omega }_{+}^{(1)}-{\omega }_{-}^{(1)}\,$ is the experimental dip splitting (indicated in Fig. 2l). This approach^53,58^ is valid because (i) the coupling strength $g$ is large and (ii) the cavity and phonon resonances match well, i.e. ${\omega }_{{{{{{\rm{cav}}}}}}}^{(1)}\approx {\omega }_{{{{{{\rm{TO}}}}}}}$ (see “Methods”). Plotting ${g}_{{{\exp }}}$ in Fig. 4a (purple symbols), good agreement with the theoretical values (blue solid line) is found, verifying that strong coupling between microcavity photons and phonons can be indeed achieved experimentally with layers of a polar material as thin as 10 nm.

### Ultrastrong coupling in fully filled cavities

**Fig. 5:**
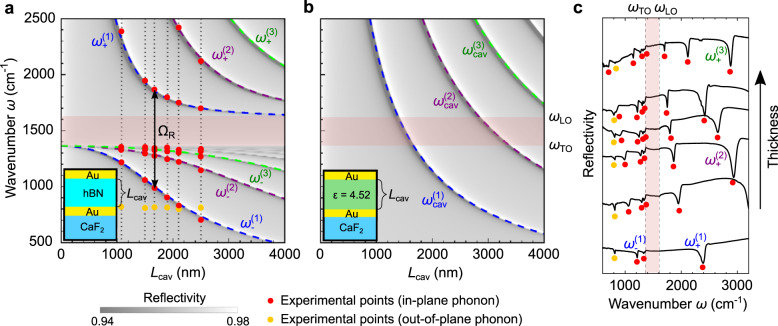
Ultrastrong coupling in cavities fully filled with hBN. **a** Reflectivity spectra of a cavity fully filled with hBN (illustrated by inset) as a function of cavity thickness ${L}_{{{{{{\rm{cav}}}}}}}$. The dashed curves show the cavities’ eigenmode frequencies $\,{\omega }_{+}^{(j)}$ and ${\omega }_{-}^{(j)}$ as a function of ${L}_{{{{{{\rm{cav}}}}}}}$. Red and yellow symbols show the spectral position of the experimental reflectivity dips extracted from panel (**c**). The reddish areas in **a** and **b** mark the Reststrahlen band of the in-plane phonon of hBN. **b** Reflectivity spectra of a bare cavity (illustrated by inset) as a function of cavity thickness ${L}_{{{{{{\rm{cav}}}}}}}$. The dashed curves show the cavities’ eigenmode frequencies $\,{\omega }_{{{{{{\rm{cav}}}}}}}^{(j)}$ as a function of ${L}_{{{{{{\rm{cav}}}}}}}$. **c** Experimental reflectivity spectra of cavities fully filled with hBN (vertically offseted). Cavity thicknesses (from bottom to top) are: 1,080 nm, 1,500 nm, 1,665 nm, 1,900 nm, 2,100 nm, 2,500 nm.

To further illustrate and explore the regime of maximum coupling strength, we performed a systematic experimental and theoretical study of cavities fully filled with hBN (Fig. 5a). Analogously to Fig. 3, we calculated reflection spectra (contour plots) and eigenmode (i.e. resonance) frequencies (dashed lines) as a function of the cavity (i.e. hBN) thickness ${L}_{{{{{{\rm{hBN}}}}}}}={L}_{{{{{{\rm{cav}}}}}}}$. By comparison with the spectra and eigenmodes of the corresponding bare cavities (Fig. 5b), we clearly recognize the anti-crossing between the hBN phonon (${\omega }_{{{{{{\rm{TO}}}}}}}$) and all cavity modes^59^ (${\omega }_{{{{{{\rm{cav}}}}}}}^{(j)}\,$with $j$ $=$ 1, 2, 3...), yielding the polaritonic eigenmodes ${\omega }_{+}^{(j)}$ and ${\omega }_{-}^{(j)}$ (contrary to Fig. 3, where coupling is observed only for odd $j$). Further, a much larger spectral separation of the upper and lower polariton branches occurs as compared to Fig. 3a (${L}_{{{{{{\rm{hBN}}}}}}}=$ 10 nm), yielding a polaritonic gap spanning the whole RB (reddish area in Fig. 5a, b). We determine a Rabi splitting of Ω_R_ $\approx$ $2g$ $=$ 856 cm^–1^, which lies well inside the ultrastrong coupling regime ($g$/${\omega }_{{{{{{\rm{TO}}}}}}}=$ 0.31), and which is larger than the RB (246 cm^–1^). We experimentally confirm the calculations by measuring reflectivity spectra of differently thick cavities that are fully filled with hBN (Fig. 5c). The spectral positions of the reflectivity dips are plotted in Fig. 5a (red symbols), showing an excellent agreement with the calculated spectra and cavity eigenmodes. We note that the experimental reflectivity spectra exhibit a set of thickness-independent dips at around 819 cm^–1^ (yellow dots in Fig. 5a), which stem from the out-of-plane phonon of hBN^32^. This phonon appears in the experimental spectra due to the focused illumination of the cavity using a Cassegrainian objective, yielding electric field components perpendicular to the hBN layer. Therefore, these field components can couple with the out-of-plane hBN phonon, as opposed to the case of normal incidence, in which the electric field can only couple with the in-plane phonon (see “Supplementary Note 4”).

## Discussion

**Fig. 6:**
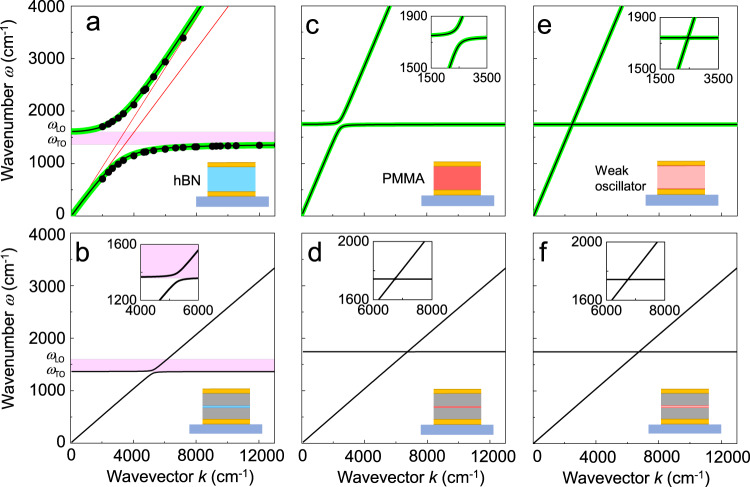
Polariton dispersions. **a** Measured (black symbols) and calculated (black curves) phonon polariton dispersion obtained from hBN layers that fully fill the cavity. Green curves show the bulk phonon polariton dispersion of hBN. Red dashed lines indicate the light cones $\omega \left(k\right)=\frac{{kc}}{\sqrt{\varepsilon (\omega =0)}}\,$ and $\left(k\right)=\frac{{kc}}{\sqrt{{\varepsilon }_{{{{{{\rm{hBN,\infty}}}}}}}}}$, where $\varepsilon (\omega =0)$ = 6.29 and ${\varepsilon }_{{{{{{\rm{hBN,\infty}}}}}}}$ = 4.52 are the low- and high-frequency permittivities. The Reststrahlen band of bulk hBN is marked by the purple area. **b** Calculated (black curves) phonon polariton dispersion obtained from cavities that are filled with a 10 nm thick hBN layer. **c** Calculated (black curves) polariton dispersion obtained for cavities that are fully filled with molecules exhibiting a C=O vibration. Green curves show the bulk polariton dispersion of the filling material. **d** Dispersion for a 10 nm thick molecular layer embedded in the cavity. **e** Calculated (black curves) polariton dispersion obtained for cavities fully filled with hypothetical molecules exhibiting a vibration of arbitrarily reduced oscillator strength. Green curves show the bulk polariton dispersion of the filling material. **f** Dispersion for a 10 nm thick hypothetical molecular layer of arbitrarily reduced coupling strength embedded in the cavity. Schematics illustrate the cavities’ cross sections.

To analyze and discuss the dispersion of the microcavity phonon polaritons, $\omega (k)$, we extracted the wavevector $k$ from the reflectivity spectra according to

1 $$k=\frac{j\pi \,}{{L}_{{{{{{\rm{cav}}}}}}}},$$

assuming perfect metal mirrors and where *L*_cav_ is the cavity length at which the eigenmode of order *j* and frequency $\omega$ is found (see “Methods”). In Fig. 6a, b we compare $\omega (k)$ for the cavity that is fully filled with hBN (obtained from Fig. 5) and the cavity embedding a 10 nm thick hBN layer (obtained from Fig. 3), respectively (calculations are shown by black curves and experimental values by black symbols). Note that for the fully filled cavity, all modes $j$ were considered, whereas for the cavity filled with 10 nm of hBN we considered only the first mode ($j=$ 1). Both dispersions feature anti-crossing, separating into a lower and an upper microcavity phonon polariton branch, with large Rabi splitting amounting to ${\Omega }_{{{{{{\rm{R}}}}}}}\approx$ 856 cm^–1^ and ${\Omega }_{{{{{{\rm{R}}}}}}}\approx$ 63 cm^–1^, respectively. To appreciate the dramatic coupling strengths between infrared microcavity modes and phonons with respect to typical molecular vibrations^50^, we show in Fig. 6c, d the calculated polariton dispersions obtained (analogous to Figs. 3 and 5) for cavities embedding molecules that possess C=O vibrations. Specifically, we consider the ensemble of C=O oscillators of poly(methyl methacrylate) (PMMA), whose electromagnetic response is described by the dielectric function provided in Supplementary Note 1. Clear anti-crossing can be observed for the cavity fully filled with PMMA (Fig. 6c), but the Rabi splitting ${\Omega }_{{{{{{\rm{R}}}}}}}\approx$ 159 cm^–1^ is more than five times smaller than that of the cavity fully filled with hBN. For the cavity filled with a 10 nm thick PMMA layer, we do not find anti-crossing (Fig. 6d), revealing that the system is in the weak coupling regime, in contrast to the strong coupling regime achieved with a 10 nm thick hBN layer (Fig. 6b). We note that the C=O vibrations are rather strong and that many molecular vibrations can be much weaker. To demonstrate the coupling between microcavity modes and weaker molecular oscillators by way of an example, we reduced the oscillator strength in the permittivity model of C=O by a factor of 100 and recalculated the dispersions. In both the partially and fully filled cavity we do not observe anti-crossing (Fig. 6e, f), highlighting that for weak molecular oscillators strong coupling cannot be achieved by placing them into a microcavity.

We finally compare the dispersions of the material in the fully filled cavities with the bulk polariton dispersion of the same material, ${\omega}\, \left(k\right)=\frac{{c\; k}}{\sqrt{{\varepsilon }_{{{{{{\rm{m}}}}}}}}}$, where $c$ is the speed of light and ${\varepsilon }_{{{{{{\rm{m}}}}}}}$ dielectric function of the filling material (green solid lines in Fig. 6a, c, and e). Interestingly, we find that the polariton dispersion obtained from fully filled cavities is identical to that of the bulk polariton dispersion of the filling material^60^, independent of whether phonon or molecule oscillators (weak or strong) are embedded into the cavity. The maximum splitting is determined exclusively by the material properties as clearly shown by the analytical expression of $g$ in the Supplementary Note 6 and is also highlighted in ref. ^35,61,62^. These results show that the maximum coupling strength between a cavity mode and a dipolar excitation is governed by that of photons and bulk, implying that fully filling a resonant cavity with a specific material does not enhance the coupling strength between light and matter. The cavity merely enforces the strongly coupled state by selecting the corresponding wavevector. Importantly, strong coupling in a cavity can be only achieved in the case that the bulk dispersion of the filling material already exhibits strong coupling, i.e. anti-crossing. In the case that the dispersion of light in the bulk material does not exhibit polaritonic behavior, the coupling of this bulk material with a cavity mode will remain weak. In other words, placing a material into a cavity will not make the coupling strength to exceed that of the bulk material. Interestingly, it has been reported that vibrational strong coupling in cavities can modify physical and chemical properties of the material filling the cavity^2,17,63,64^. Since the coupling strength is not enhanced, another effect - such as a modification of the density of states - may be needed to explain this intriguing phenomenon.

In conclusion, we demonstrated that classical microcavities can be applied for studying and tuning the coupling between photons and optical phonons in thin layers of polar vdW materials, which can be obtained in high crystal quality by exfoliation. For the studied vdW material hBN, our theoretical analysis predicts strong coupling for hBN layers between 3 and 148 nm thickness and ultrastrong coupling for hBN thicknesses larger than 148 nm. Analysis of experimental reflection spectra of cavities embedding 10 and 100 nm thick hBN layers confirms strong coupling. For fully filled cavities ultrastrong was demonstrated experimentally. In comparison with typical molecular vibrational strong coupling in fully filled cavities, the coupling strength is about five times larger due to the high oscillator strength of crystal phonons. Our experiments can be readily adapted to study phonon polaritons in other vdW materials^65^, including doped semiconductors and heterostructures exhibiting multiple phonons or plasmon–phonon coupling. Further, by using vdW materials with even larger oscillator strength than hBN, the deep strong coupling regime^66–68^ may be reached. We also note that recent experimental reports have demonstrated the possibility of tuning optical phonon frequencies by various methods, such as photoinjection^69^, heterostructuring^70^, and intercalation^71^, which could allow for manipulating the phonon polariton spectrum, similarly to intersubband polaritonic systems^72^. Considering the intriguing vibrational strong-coupling phenomena that have been reported in literature it may be interesting to study how ultrastrong coupling of phonons may affect physical and chemical properties of crystals that are embedded into cavities. Only recently it has been predicted that strong coupling in quantum paraelectrics can trigger the ferroelectric phase^16^.

## Methods

### Fabrication of fully filled microcavities

First, the bottom optical mirror was prepared on a CaF2 substrate by thermally evaporating 20 nm of gold. The evaporation was performed in high vacuum conditions (pressure 10^–6^ mbar) at a rate of 0.8 nm/min. The reflectivity of this mirror, *R*, was measured by FTIR spectroscopy, yielding *R* = 0.98 for the spectral range studied in this work.

Large thick flakes of hexagonal Boron Nitride (hBN) were obtained via mechanical exfoliation of commercially available hBN crystals (HQ Graphene Co.) using blue Nitto tape (Nitto SPV 224P). Subsequently, the blue Nitto tape was placed on a polydimethylsiloxane (Gelpack PF GEL film WF 4, 17 mil.) transparent stamp. This way, after removing the blue Nitto tape from the polydimethylsiloxane stamp, some flakes remain on the polydimethylsiloxane stamp. Flakes of approximately the desired thickness were optically identified. The chosen ones were transferred on top of the bottom optical mirror using the deterministic dry transfer technique^51^. In order to characterize the thicknesses of the hBN flakes, we used a profilometer (Dektak 150), which has an experimental measurement accuracy of about 5%. Large areas of the aimed thickness (ranging from 1,000 to 2,500 nm) were consequently identified and localized.

Finally, the sample was covered again with 20 nm of thermally evaporated gold, to form the top optical mirror. This evaporation was performed under the same conditions as for the bottom optical mirror.

### Fabrication of microcavities embedding thin layers of hBN

Using the same techniques as for the case of hBN flakes, flakes of MoS_2_ were exfoliated from MoS_2_ crystals (SPI supplies). The flakes were characterized and transferred onto a bottom optical mirror, prepared as in the previous sample (20 nm of gold thermally evaporated on a CF2 substrate). Thin hBN flakes were obtained and optically identified, then transferred on the MoS_2_ flake already on the gold layer. The heterostructure thickness was measured with the profilometer. A second MoS_2_ flake of ideally the same thickness as the first one was transferred on top of the MoS_2_-hBN structure. In order to obtain the final thickness of the stack, it was once again characterized with the profilometer. A second layer of gold 20 nm thick was then thermally evaporated over the structure.

### Determination of the coupling strength $g$ in TM simulations

To determine the coupling strength $g$ in the TM simulations, we modeled the phonon–photon interaction by the coupling of two classical harmonic oscillators, where one of the oscillators is associated with the electromagnetic field of the cavity and the other one with the hBN phonon (Supplementary Note 2.3). For small $g$ (corresponding to the weak and strong coupling regimes), the eigenfrequencies of the polaritonic modes, which correspond to the eigenvalues of the harmonic oscillator model, are given by

2 $${\omega }_{\pm }^{\left(j\right)}=\frac{1}{2}\left({\omega }_{{{{{{\rm{cav}}}}}}}^{\left(j\right)}+{\omega }_{{{{{{\rm{TO}}}}}}}\right)\pm \frac{1}{2}{Re}\left[\sqrt{{\left({\omega }_{{{{{{\rm{cav}}}}}}}^{\left(j\right)}-{\omega }_{{{{{{\rm{TO}}}}}}}+i\frac{\gamma -\kappa }{2}\right)}^{2}+4{g}^{2}}\right],$$

where $\kappa$ and $\gamma$ are the cavity and phonon decay rates, respectively. For large $g$ (corresponding to the ultrastrong coupling regime), we can neglect the losses $\kappa$ and $\gamma$ and obtain the following expression from the equations of the coupled harmonic oscillators:

3 $${\omega }_{\pm }^{\left(j\right)}=\frac{1}{\sqrt{2}}\sqrt{{\left({\omega }_{{{{{{\rm{cav}}}}}}}^{\left(j\right)}\right)}^{2}+{\omega }_{{{{{{\rm{TO}}}}}}}^{2}+4{g}^{2}\pm \sqrt{{\left({\left({\omega }_{{{{{{\rm{cav}}}}}}}^{\left(j\right)}\right)}^{2}+{\omega }_{{{{{{\rm{TO}}}}}}}^{2}+4{g}^{2}\right)}^{2}-4{\left({\omega }_{{{{{{\rm{cav}}}}}}}^{\left(j\right)}\right)}^{2}{\omega }_{{{{{{\rm{TO}}}}}}}^{2}}}.$$

The eigenfrequencies given by Eq. (3) coincide with the results obtained from the Hopfield Hamiltonian, indicating that a classical model of harmonic oscillators can describe the ultrastrong coupling regime^35^ (Supplementary Note 2.3). For intermediate values of $g$, Eq. (2) and Eq. (3) give very similar results.

In order to determine $g$ for a fixed hBN layer thickness ${L}_{{{{{{\rm{hBN}}}}}}}$, we choose the total thickness ${L}_{{{{{{\rm{cav}}}}}}}$ for which the cavity mode is tuned to the TO phonon (i.e. ${\omega }_{{{{{{\rm{cav}}}}}}}^{(j)}$ $=$ ${\omega }_{{{{{{\rm{TO}}}}}}}$ $=$ 1,364 cm^–^^1^). Then, we determine for this cavity the eigenfrequencies of the modes (${\omega }_{+}^{\left(j\right)}$ and ${\omega }_{-}^{\left(j\right)}$ for the filled cavity, and ${\omega }_{{{{{{\rm{cav}}}}}}}^{\left(j\right)}$ for the bare cavity) from the poles of the reflection coefficient of the system, as obtained from the TM simulations^73^ (for details see “Supplementary Note 2”). We note that the spectral position of reflection minima and the actual polaritonic modes of a system can be different due to the interference of spectrally closely spaced modes (particularly in weakly coupled systems^5,53^). The coupling strength is finally obtained by solving Eqs. (2) or (3) for $g$ (depending on whether the system is in the strong or in the ultrastrong coupling regime). Note that the value of $g$ varies slightly depending on whether we solve the equations for ${\omega }_{+}^{\left(j\right)}$ or ${\omega }_{-}^{\left(j\right)}$. We thus determine $g$ by minimizing the combined error.

From the TM simulations considering a 10 nm hBN layer in the middle of the cavity (Fig. 3a) we obtained ${\omega }_{+}^{\left(1\right)}$ $=$ 1,395 cm^–^^1^, ${\omega }_{-}^{\left(1\right)}=$ 1,332 cm^–^^1^, $\kappa =$ 60 cm^–^^1^, and $\gamma$ $=$ 5 cm^–^^1^, yielding subsequently $g$ ≈ 34 cm^–^^1^ via the harmonic oscillator model. For the 100 nm hBN layer (Supplementary Note 3) we obtained ${\omega }_{+}^{\left(1\right)}$ $=$ 1,471 cm^–^^1^, ${\omega }_{-}^{\left(1\right)}=$ 1,260 cm^–^^1^, $\kappa =$ 60 cm^–^^1^, $\gamma$ $=$ 5 cm^–^^1^, and subsequently $g$ ≈ 106 cm^–^^1^. For the fully filled cavity (Fig. 5a) we obtained ${\omega }_{+}^{\left(1\right)}=$ 1,857 cm^–^^1^, ${\omega }_{-}^{\left(1\right)}=$ 1,002 cm^–^^1^, $\kappa =$ 30 cm^–^^1^, $\gamma$ $=$ 5 cm^–^^1^, and subsequently $g$ ≈ 428 cm^–^^1^. We note that the values for ${\omega }_{+}^{\left(1\right)}$ and ${\omega }_{-}^{\left(1\right)}$ obtained for these three cavities are very close to the frequencies of the calculated reflectivity dips. For example, for the cavity containing the 10 nm hBN layer, the reflection dips occur at ${\omega }_{+}^{\left(1\right)}$ = 1,392 cm^–^^1^ and ${\omega }_{-}^{\left(1\right)}=$ 1,322 cm^–^^1^. We conclude that the dips in the experimental reflection spectra reveal the polaritonic eigenmodes and thus can be directly used to determine the experimental coupling strengths.

### Comparison between phonon polariton dispersions

The polaritonic modes in bulk hBN are associated with propagating waves characterized by a wavevector $k$ and an angular frequency $\omega$ that are connected according to the dispersion relation $\omega (k)$. On the other hand, the modes of the cavity for normal incidence are standing waves with selected discrete wavelengths ${\lambda }_{{{{{{\rm{cav}}}}}}}$, which depend on the cavity length ${L}_{{{{{{\rm{cav}}}}}}}$, according to ${\lambda }_{{{{{{\rm{cav}}}}}}}=\frac{{2{L}}_{{{{{{\rm{cav}}}}}}}}{j}$ ($j$ is the order of the mode). For the fully filled cavity, ${\lambda }_{{{{{{\rm{cav}}}}}}}$ would also correspond to the wavelength of the phonon polariton in bulk. Thus, for a given resonant frequency $\omega$, we recover the corresponding wavevector *k* of the bulk dispersion relation $\omega (k)$ by applying $k=\frac{2\pi }{{\lambda }_{{{{{{\rm{cav}}}}}}}}=\frac{j\pi }{{L}_{{{{{{\rm{cav}}}}}}}}$.

## Supplementary information

Supplementary Information

## Data Availability

Data that support the results of this work are available upon reasonable request from the corresponding author (r.hillenbrand@nanogune.eu).
