# Supplementary material for: Microcavity phonon polaritons from the weak to the ultrastrong phonon–photon coupling regime
Source: Nat Commun. 2021 Oct 27;12:6206. doi: 10.1038/s41467-021-26060-x (PMC8551273; doi:10.1038/s41467-021-26060-x)
Supplement: Supplementary file 1 — Supplementary Information [file 41467_2021_26060_MOESM1_ESM.pdf]

Supplementary Information for: Microcavity phonon polaritons from the weak to the ultrastrong phonon-photon coupling regime

María Barra-Burillo[1], Unai Muniain[2], Sara Catalano[1], Marta Autore[1], Felix Casanova[1,3], Luis E. Hueso[1,3], Javier Aizpurua[2,4], Ruben Esteban[2,4] and Rainer Hillenbrand[3,5]

[1] *CIC nanoGUNE BRTA, 20018 Donostia-San Sebastián, Spain*

[2] *Donostia International Physics Center, 20018 Donostia-San Sebastián, Spain*

[3] *IKERBASQUE, Basque Foundation for Science, 45011 Bilbao, Spain*

[4] *Materials Physics Center, CSIC-UPV/EHU, 20018 Donostia-San Sebastián, Spain*

[5] *CIC nanoGUNE BRTA and Department of Electricity and Electronics, EHU/UPV, 20018 Donostia-San Sebastián, Spain*

Supplementary Notes:

1 Description of the cavities and permittivities of the different materials 2

2 Characterization of the polaritonic frequencies 4

2.1 Transfer-matrix simulations 4

2.2 Frequencies of the modes 6

2.3 Coupled harmonic oscillator model 6

3 Reflectivity spectra of the cavity embedding a 100 nm-thick hBN layer 10

4 Reflectivity of the system under focused illumination 10

5 Angle dependence of the reflectivity spectra 13

6 Analytical expression for the coupling strength 14

6.1 Coupling strength of a TO phonon with a Fabry-Pérot cavity mode 14

6.2 Application to the hBN microcavities 16

7 Dependence of the coupling strength on the hBN layer position 19

1 Description of the cavities and permittivities of the different materials

In this Supplementary Note, we describe in more detail the hexagonal boron nitride (hBN) microcavities that we analyze in this work, including all the different thicknesses. We use Fabry-Pérot cavities containing hBN layers of variable thickness. A schematic diagram is shown in Supplementary Fig. 1. The cavities are formed by planar layers, and all the interfaces are perpendicular to the z axis, as shown by the coordinates axes included in the scheme. In all the calculations the incident medium (i.e. the medium from which the system is illuminated) is vacuum, the substrate is CaF_2 and the mirrors are 20 nm-thick gold layers. The inside of the cavity, i.e. without considering the mirrors, extends from $z = 0$ to $z = L_{\mathrm{cav}}$ (L_{cav} is the total thickness), and it contains a layer of thickness L_{hBN} placed between $z = L_1$ and $z = L_2$. In all the experiments and in many of the transfer-matrix simulations, the material of this layer is hBN, and we refer to this particular case as a *hBN-filled cavity*. In other simulations, we replace hBN by a material with constant permittivity $\varepsilon_{\mathrm{hBN},\infty}$, which corresponds to the high-frequency permittivity of hBN (see below). We refer to these cavities as *bare cavities*, because the contribution of the hBN phonons is eliminated. The rest of the cavity (the layers between $z = 0$ and $z = L_1$ and between $z = L_2$ and $z = L_{\mathrm{cav}}$) is filled by MoS_2, which does not show any resonant feature in the analyzed range of frequencies.

In the transfer-matrix simulations, we have modelled the materials of the system as follows. For the incident medium we use the vacuum permittivity, and the relative permittivity of the substrate material CaF_2 is $\varepsilon_{\mathrm{CaF}_2} = 1.882$. The gold mirrors are described by a Drude permittivity that fits the low-frequency experimental data from Supplementary Ref. [1]:

$$\varepsilon_{\mathrm{Au}}(\omega) = 1 - \frac{\omega_{\mathrm{p,Au}}^2}{\omega(\omega + i\gamma_{\mathrm{Au}})}, \tag{1}$$

with plasma frequency $\omega_{\mathrm{p,Au}} = 73114.15$ cm^{-1} and damping frequency $\gamma_{\mathrm{Au}} = 571.04$ cm^{-1} [2].

We model hBN with a diagonal permittivity tensor $\overleftrightarrow{\varepsilon}_{\mathrm{hBN}}(\omega)$, in order to take into account the anisotropy of this material. hBN is characterized by a layered atomic structure, so that the phonons that are polarized along the plane of the atomic layers (in-plane phonons) have different frequencies compared to those polarized in the normal direction, i.e. parallel to the anisotropic axis ($\|$, out-of-plane phonons). In our configuration, the plane of the atomic layers is parallel to all the Fabry-Pérot interfaces ($x - y$ plane). We thus have $\overleftrightarrow{\varepsilon}_{\mathrm{hBN}}(\omega) = \mathrm{diag}\left(\varepsilon_{\mathrm{hBN}}(\omega), \varepsilon_{\mathrm{hBN}}(\omega), \varepsilon_{\mathrm{hBN},\|}(\omega)\right)$, where we omit the second subscript in the x and y components for brevity (notice that the x and y components are identical). In particular, the in-plane permittivity tensor component $\varepsilon_{\mathrm{hBN}}(\omega)$ follows a Lorentzian function [3]:

$$\varepsilon_{\mathrm{hBN}}(\omega) = \varepsilon_{\mathrm{hBN},\infty}\left(1 + \frac{\omega_{\mathrm{LO}}^2 - \omega_{\mathrm{TO}}^2}{\omega_{\mathrm{TO}}^2 - \omega^2 - i\omega\gamma}\right), \tag{2}$$

where ω_{TO} is the corresponding transverse optical (TO) phonon frequency, ω_{LO} is the longitudinal optical (LO) phonon frequency, γ is the damping frequency and $\varepsilon_{\mathrm{hBN},\infty}$ is the high-frequency permittivity. For our analysis, the exact value of ω_{TO} determines the detuning between the cavity mode and the phonon, which is a crucial parameter in strong coupling. Therefore, to obtain the value of this parameter in our samples, we measured the reflectivity spectra of a 100 nm-thick hBN layer before the fabrication of the cavities. A clear resonance was observed at $\omega_{\mathrm{TO}} = 1364$ cm^{-1}, which is the value that we consider in the simulations. This frequency is very close to the value $\omega_{\mathrm{TO}} = 1360$ cm^{-1}

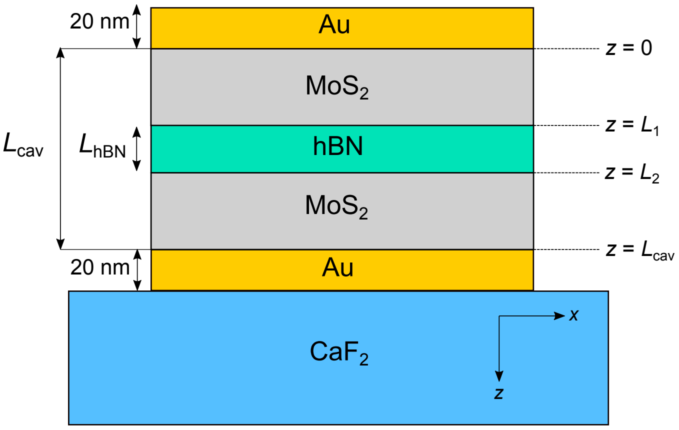

Supplementary Figure 1: Schematic diagram of the hBN microcavities. We indicate the coordinates axes and the material and thickness of each layer. In some simulations, hBN is substituted by PMMA, a weak oscillator or a material with constant permittivity $\varepsilon_{\mathrm{hBN},\infty}$.

reported in Supplementary Ref. [3]. For all the other parameters in Supplementary Eq. (2) we use the values of Supplementary Ref. [3]: $\varepsilon_{\mathrm{hBN},\infty} = 4.52$, $\omega_{\mathrm{LO}} = 1610$ cm^{-1} and $\gamma = 5$ cm^{-1}.

Supplementary equation (2) properly describes the optical response of hBN in all the results in the main text, because the electric fields are directed along the x direction and thus hBN can be treated as an isotropic material with the permittivity given by Supplementary Eq. (2). This approach is also followed in the Supplementary Information when not stated otherwise. However, in Supplementary Note 4 we consider focused illumination and in Supplementary Note 5 we analyze the response of the system as a function of the incident angle, where it is necessary to take the anisotropy of hBN into account. In this case, the permittivity tensor component along the z axis $\varepsilon_{\mathrm{hBN},||}(\omega)$ follows the same Lorentzian function as in Supplementary Eq. (2), except that the values of the parameters are $\varepsilon_{\mathrm{hBN},\infty} = 4.52$, $\omega_{\mathrm{TO},||} = 746$ cm^{-1}, $\omega_{\mathrm{LO},||} = 819$ cm^{-1} and $\gamma_{||} = 4$ cm^{-1} (taken from Supplementary Ref. [3]).

In order to obtain the permittivity of MoS_2, we have fabricated three microcavities fully filled by MoS_2 of different nominal thickness: $L_{\mathrm{cav}} = 660$ nm, 740 nm and 960 nm. The geometry is the same as in Supplementary Fig. 1 but without the hBN layer. We measure the reflectivity spectra and obtain the frequencies of the Fabry-Pérot modes for each cavity up to 8000 cm^{-1}. We then perform transfer-matrix simulations of the cavities and find the value of the permittivity $\varepsilon_{\mathrm{MoS}_2}$ required to reproduce the position of the experimental dips. We show in Supplementary Fig. 2 the frequencies of the dips for the three cavities and their corresponding value of $\varepsilon_{\mathrm{MoS}_2}$. A linear fitting of these results (dashed line) gives the permittivity that we use in the simulations:

$$\varepsilon_{\mathrm{MoS}_2}(\omega) = 14.689 + 0.000151\omega, \tag{3}$$

where ω is in units of cm^{-1}.

Last, in some simulations in the end of the main text, we replace hBN with PMMA and a weak oscillator. For PMMA, we use a permittivity that also follows a Lorentzian form [4]:

$$\varepsilon_{\mathrm{PMMA}}(\omega) = \varepsilon_{\mathrm{PMMA},\infty} + F_{\mathrm{PMMA}} \frac{\omega_{\mathrm{PMMA}}^2}{\omega_{\mathrm{PMMA}}^2 - \omega^2 - i\omega\gamma_{\mathrm{PMMA}}}, \tag{4}$$

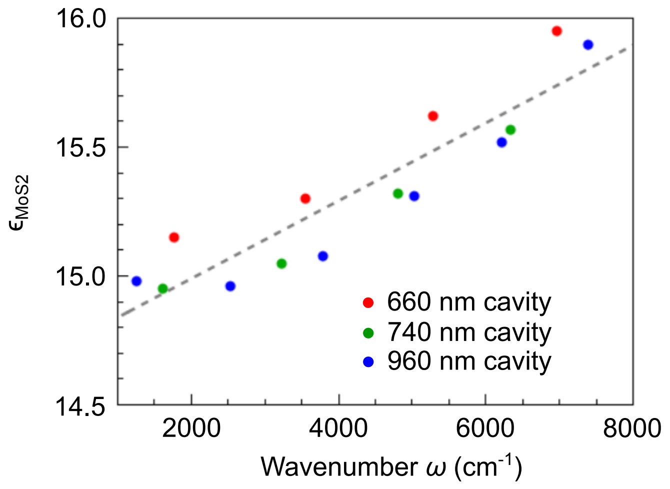

Supplementary Figure 2: Linear fitting of the permittivity of MoS_2. The dots indicate the experimental resonant wavenumber of the different Fabry-Pérot modes and the corresponding permittivity extracted from these values according to the procedure described in the text, for three cavities fully filled by MoS_2 of nominal thickness $L_{\rm cav} = 660$ nm (red dots), $L_{\rm cav} = 740$ nm (green dots) and $L_{\rm cav} = 960$ nm (blue dots). The dashed line corresponds to the linear fitting of all dots, given by Supplementary Eq. (3).

with high-frequency permittivity $\varepsilon_{\rm PMMA,\infty} = 1.99$, vibrational frequency $\omega_{\rm PMMA} = 1742$ cm^{-1}, damping frequency $\gamma_{\rm PMMA} = 13$ cm^{-1} and oscillator strength $F_{\rm PMMA} = 0.0165$. When we consider a weak oscillator in Figs. 6e and 6f in the main text, we use the same values as for PMMA, except for $F_{\rm PMMA} = 1.65 \cdot 10^{-4}$.

2 Characterization of the polaritonic frequencies

2.1 Transfer-matrix simulations

We first summarize briefly how to calculate the reflectivity spectra of hBN microcavities using the transfer-matrix formalism [5]. This method allows to obtain the total reflection $r^{s(p)}_{\rm total}$ and transmission $t^{s(p)}_{\rm total}$ coefficients of a $s(p)$-polarized planewave, for any layered structure, in terms of the thickness d_i and relative permittivity ε_i of each layer i and the Fresnel coefficients $r^{s(p)}_{i,i+1}$ and $t^{s(p)}_{i,i+1}$ of each interface between layers i and $i+1$. In total, the system contains $N_{\rm lay}$ finite-sized layers, and we label the incident medium as $i = 0$ and the substrate as $i = N_{\rm lay} + 1$. For the calculations in the main text, we consider that the light is incident along the z direction perpendicular to the planar interfaces and all materials are considered isotropic. However, for the calculations in Supplementary Notes 4 and 5, we analyze the effects of the anisotropy of hBN and, thus, here we discuss the complete formalism for anisotropic materials. To take the anisotropy of hBN into account, we consider that each layer i has a permittivity tensor of the general diagonal form $\overset{\leftrightarrow}{\varepsilon_i} = {\rm diag}(\varepsilon_{i,x}, \varepsilon_{i,y}, \varepsilon_{i,z})$ ($\varepsilon_{{\rm hBN},x} = \varepsilon_{{\rm hBN},y}$ for hBN in our experiments). Light is incident in the $x - z$ plane. The Fresnel coefficients are given by [5]

$$r^s_{i,i+1} = \frac{\sqrt{\varepsilon_{i,y} - \xi^2} - \sqrt{\varepsilon_{i+1,y} - \xi^2}}{\sqrt{\varepsilon_{i,y} - \xi^2} + \sqrt{\varepsilon_{i+1,y} - \xi^2}}, \qquad r^p_{i,i+1} = \frac{\varepsilon_{i+1,x}\sqrt{\varepsilon_{i,x}\left(1 - \frac{\xi^2}{\varepsilon_{i,z}}\right)} - \varepsilon_{i,x}\sqrt{\varepsilon_{i+1,x}\left(1 - \frac{\xi^2}{\varepsilon_{i+1,z}}\right)}}{\varepsilon_{i+1,x}\sqrt{\varepsilon_{i,x}\left(1 - \frac{\xi^2}{\varepsilon_{i,z}}\right)} + \varepsilon_{i,x}\sqrt{\varepsilon_{i+1,x}\left(1 - \frac{\xi^2}{\varepsilon_{i+1,z}}\right)}},$$

$$t^s_{i,i+1} = \frac{2\sqrt{\varepsilon_{i,y} - \xi^2}}{\sqrt{\varepsilon_{i,y} - \xi^2} + \sqrt{\varepsilon_{i+1,y} - \xi^2}}, \qquad t^p_{i,i+1} = \frac{2\varepsilon_{i+1,x}\sqrt{\varepsilon_{i,x}\left(1 - \frac{\xi^2}{\varepsilon_{i,z}}\right)}}{\varepsilon_{i+1,x}\sqrt{\varepsilon_{i,x}\left(1 - \frac{\xi^2}{\varepsilon_{i,z}}\right)} + \varepsilon_{i,x}\sqrt{\varepsilon_{i+1,x}\left(1 - \frac{\xi^2}{\varepsilon_{i+1,z}}\right)}}, \tag{5}$$

where $\xi = k_x/k_0$ is the parallel component of the wavevector k_x normalized with respect to the wavevector in vacuum k_0.

The total transfer matrix $\mathbf{T}^{s(p)}$ relates the amplitudes of the electromagnetic field in the incident medium ($i = 0$) and the substrate ($i = N_{\text{lay}}+1$) for $s(p)$-polarized light: $\begin{pmatrix} A^{s(p)}_{0,+} \\ A^{s(p)}_{0,-} \end{pmatrix} = \mathbf{T}^{s(p)} \begin{pmatrix} A^{s(p)}_{N_{\text{lay}}+1,+} \\ A^{s(p)}_{N_{\text{lay}}+1,-} \end{pmatrix}$. $A^s_{i,\pm}$ and $A^p_{i,\pm}$ correspond to the amplitude of the electric and magnetic field, respectively (the amplitude of the field parallel to the interface for each polarization). The second subindex indicates the direction of propagation. + corresponds to the direction of the incoming planewave (and thus also to the direction of the transmitted light) and, similarly, the subindex - corresponds to the direction of the reflected light. In our system, $A^{s(p)}_{N_{\text{lay}}+1,-} = 0$, because in the substrate the electromagnetic field only propagates in the direction of the transmission. $\mathbf{T}^{s(p)}$ is given by the expression

$$\mathbf{T}^{s(p)} = \mathbf{M}^{s(p)}_{0,1}\mathbf{P}^{s(p)}_1\mathbf{M}^{s(p)}_{1,2}\mathbf{P}^{s(p)}_2\mathbf{M}^{s(p)}_{2,3}...\mathbf{M}^{s(p)}_{N_{\text{lay}}-1,N_{\text{lay}}}\mathbf{P}^{s(p)}_{N_{\text{lay}}}\mathbf{M}^{s(p)}_{N_{\text{lay}},N_{\text{lay}}+1}, \tag{6}$$

with

$$\mathbf{M}^{s(p)}_{i,i+1} = \frac{1}{t^{s(p)}_{i,i+1}} \begin{pmatrix} 1 & -r^{s(p)}_{i,i+1} \\ -r^{s(p)}_{i,i+1} & 1 \end{pmatrix}, \tag{7}$$

$$\mathbf{P}^s_i = \begin{pmatrix} e^{-i\frac{\omega}{c}d_i\sqrt{\varepsilon_{i,y}-\xi^2}} & 0 \\ 0 & e^{i\frac{\omega}{c}d_i\sqrt{\varepsilon_{i,y}-\xi^2}} \end{pmatrix}, \qquad \mathbf{P}^p_i = \begin{pmatrix} e^{-i\frac{\omega}{c}d_i\sqrt{\varepsilon_{i,x}\left(1-\frac{\xi^2}{\varepsilon_{i,z}}\right)}} & 0 \\ 0 & e^{i\frac{\omega}{c}d_i\sqrt{\varepsilon_{i,x}\left(1-\frac{\xi^2}{\varepsilon_{i,z}}\right)}} \end{pmatrix}. \tag{8}$$

The matrix $\mathbf{M}^{s(p)}_{i,i+1}$ relates the amplitudes of the electromagnetic field propagating both in the + and − directions, at both sides of the interface between layers i and $i + 1$. $\mathbf{P}^{s(p)}_i$ describes the propagation of light through the layer i. Once the total matrix $\mathbf{T}^{s(p)} = \begin{pmatrix} T^{s(p)}_{11} & T^{s(p)}_{12} \\ T^{s(p)}_{21} & T^{s(p)}_{22} \end{pmatrix}$ is obtained, these matrix elements are used to calculate the total transmission and reflection coefficients of the system as $t^{s(p)}_{\text{total}} = \frac{A^{s(p)}_{N_{\text{lay}}+1,+}}{A^{s(p)}_{0,+}} = \frac{1}{T^{s(p)}_{11}}$ and $r^{s(p)}_{\text{total}} = \frac{A^{s(p)}_{0,-}}{A^{s(p)}_{0,+}} = \frac{T^{s(p)}_{21}}{T^{s(p)}_{11}}$.

Last, we obtain the reflectivity spectra $\mathcal{R}^{s(p)}$, which is defined as the ratio between the intensity of the reflected and incident light. It is obtained as $\mathcal{R}^{s(p)} = |r^{s(p)}_{\text{total}}|^2$ ($r^{s(p)}_{\text{total}}$ corresponds to the ratio of the amplitude of the electromagnetic field). Once $r^{s(p)}_{\text{total}}$ and $t^{s(p)}_{\text{total}}$ are known, this approach can also be used to calculate the electromagnetic fields in any point of space. We stress that, as discussed above, in every transfer-matrix simulation shown in this work except in Supplementary Notes 4 and 5, we consider light incident in the z direction ($\xi = 0$). Under this condition, the z component of the permittivity does not affect the results and, taking into account that $\varepsilon_{\text{hBN},x} = \varepsilon_{\text{hBN},y}$ for hBN, we recover the usual equations for isotropic materials. The anisotropy needs to be considered in Supplementary Note 4 for focused illumination and in Supplementary Note 5 for oblique-incident illumination.

2.2 Frequencies of the modes

The coupling strength g is determined from the position of the eigenfrequencies of the system. In general, however, the reflectivity dips (or transmission peaks) that are found in experimental optical spectra do not need to coincide with the eigenfrequencies, particularly for small coupling strengths. To characterize these complex eigenfrequencies, we also use the transfer-matrix formalism. From the total transfer matrix $\mathbf{T}^{s(p)}$, we obtain the analytical expression for the reflection and transmission coefficients $r^{s(p)}_{\text{total}}$ and $t^{s(p)}_{\text{total}}$ of the total system. Then, we solve numerically the complex frequencies for which the denominator of these coefficients vanishes (i.e. the poles), by extending all the analytical expressions for the permittivities (see Supplementary Note 1) to complex values of ω.[a] The eigenfrequencies of the bare-cavity modes and of the polaritonic modes correspond to the complex poles of the reflection and transmission coefficients of the bare and hBN-filled cavities, respectively. The real part of these eigenfrequencies is the mode frequency and the imaginary part is equal to (minus) half the losses of the mode.

We compare in Supplementary Fig. 4 and in Figs. 3 and 5 of the main text the real part of these eigenfrequencies (indicated by dashed lines) with the frequencies of the reflectivity dips for the three cavities explored in more detail in this work (100 nm-thick hBN layer, 10 nm-thick hBN layer and the fully filled cavity, respectively). The agreement between these two frequencies is very good, and thus in these systems the reflectivity dips are a good indication of the bare-cavity and polaritonic modes. However, the calculation of the eigenfrequencies is important for determining the coupling strength for very thin layers in Fig. 4 of the main text.

2.3 Coupled harmonic oscillator model

In this subsection, we explain the procedure that we follow in the main text to compute the coupling strength g. This method is based on modelling the interaction between the material and the cavity mode by means of two coupled harmonic oscillators. This discussion is complemented in Supplementary Note 6 with an alternative method to calculate g, where we derive an analytical expression based on the microscopic interaction of the phononic material with the electric field of the cavity mode.

In this model, one oscillator ($x^{(j)}_{\text{cav}}$) is associated to the electromagnetic field of the mode of order j and frequency $\omega^{(j)}_{\text{cav}}$ of the bare cavity, a cavity where we eliminate the phonon contribution and describe the permittivity of hBN as $\varepsilon_{\text{hBN},\infty}$. The second ($x_{\text{TO}}$) represents the phonon at frequency ω_{TO} (which is modelled in Supplementary Note 6 explicitly as the collective excitation of the dipoles associated with the atomic vibrations at each unit cell within hBN). The coupling strength between these two oscillators is g, and the equations of motion are [6–9]:

$$\ddot{x}^{(j)}_{\text{cav}} + \kappa\dot{x}^{(j)}_{\text{cav}} + (\omega^{(j)}_{\text{cav}})^2 x^{(j)}_{\text{cav}} - 2g\dot{x}_{\text{TO}} = 0 \tag{9a}$$

$$\ddot{x}_{\text{TO}} + \gamma\dot{x}_{\text{TO}} + \omega^2_{\text{TO}} x_{\text{TO}} + 2g\dot{x}^{(j)}_{\text{cav}} = 0. \tag{9b}$$

κ and γ are the losses of the cavity and the phonon, respectively, and the dot $\dot{}$ denotes the time derivative. We note that we use the derivative of x_{TO} and $x^{(j)}_{\text{cav}}$ in the terms describing the coupling (last term in the left hand side of both equations). This choice is necessary to reproduce the results obtained in the ultrastrong coupling regime with transfer-matrix simulations and experimentally [9].

[a]As we use simple Lorentzian and Drude functions, the expression of the permittivities can be directly evaluated at complex frequencies.

$\gamma = 5$ cm^{-1} and $\omega_{\rm TO} = 1364$ cm^{-1} are taken from the hBN dielectric function (Supplementary Note 1) and $\omega_{\rm cav}^{(j)}$ and κ are extracted from the complex pole $\omega_{\rm cav}^{(j)} - i\frac{\kappa}{2}$ of the reflection and transmission coefficients of the bare cavity (these eigenvalues are obtained as explained in Supplementary Note 2.2). In our system, the losses of the cavity vary between $\kappa \approx 30$ cm^{-1}, for the cavity fully filled with hBN, and $\kappa \approx 60$ cm^{-1}, for cavities with very thin layers of hBN. These values yield quality factors $Q = \frac{\omega_{\rm cav}^{(j)}}{\kappa}$ between $Q \approx 22$ and $Q \approx 45$ for all cavities. On the other hand, the experimental measurement of this value κ is challenging. However, by comparing the widths of the measured reflectivity polaritonic dips with the simulations, we observe that the experimental widths are only slightly larger, and we estimate that the difference between the experimental and theoretical value of κ is at most 10% (notice that the sum of the widths of the two polaritonic dips should be close to $\kappa + \gamma$ [10]).

Supplementary equation (9) can be alternatively written in frequency domain:

$$\left[-\omega^2 - i\kappa\omega + (\omega_{\rm cav}^{(j)})^2\right] x_{\rm cav}^{(j)} + 2ig\omega\, x_{\rm TO} = 0 \tag{10a}$$

$$-2ig\omega\, x_{\rm cav}^{(j)} + \left[-\omega^2 - i\gamma\omega + \omega_{\rm TO}^2\right] x_{\rm TO} = 0. \tag{10b}$$

The polaritonic frequencies $\omega_{\pm}^{(j)}$ correspond to the values that cancel the determinant of this system of equations, i.e.,

$$\left[-(\omega_{\pm}^{(j)})^2 + (\omega_{\rm cav}^{(j)})^2 - i\kappa\omega_{\pm}^{(j)}\right]\left[-(\omega_{\pm}^{(j)})^2 + (\omega_{\rm TO}^{(j)})^2 - i\gamma\omega_{\pm}^{(j)}\right] - 4g^2(\omega_{\pm}^{(j)})^2 = 0, \tag{11}$$

which can be solved numerically for any value of the coupling strength. Further, approximate analytical solutions can be obtained for two typical situations. For small values of g, the polaritonic frequencies are similar to the bare frequencies: $\omega_{\pm}^{(j)} \approx \omega_{\rm cav}^{(j)}, \omega_{\rm TO}$. Thus, we can make the secular approximation $(\omega_{\rm cav}^{(j)})^2 - (\omega_{\pm}^{(j)})^2 = \left[\omega_{\rm cav}^{(j)} + \omega_{\pm}^{(j)}\right]\left[\omega_{\rm cav}^{(j)} - \omega_{\pm}^{(j)}\right] \approx 2\omega_{\pm}^{(j)}\left[\omega_{\rm cav}^{(j)} - \omega_{\pm}^{(j)}\right]$ (and the corresponding approximation for $\omega_{\rm TO}$) [11]. Then, Supplementary Eq. (10) becomes

$$\left[-\omega - i\frac{\kappa}{2} + \omega_{\rm cav}^{(j)}\right] x_{\rm cav}^{(j)} + ig\, x_{\rm TO} = 0 \tag{12a}$$

$$-ig\, x_{\rm cav}^{(j)} + \left[-\omega - i\frac{\gamma}{2} + \omega_{\rm TO}\right] x_{\rm TO} = 0, \tag{12b}$$

and Supplementary Eq. (11) becomes quadratic in $\omega_{\pm}^{(j)}$, with solutions [3]

$$\omega_{\pm}^{(j)} = \frac{1}{2}(\omega_{\rm cav}^{(j)} + \omega_{\rm TO}) \pm \frac{1}{2}\,{\rm Re}\left[\sqrt{\left(\omega_{\rm cav}^{(j)} - \omega_{\rm TO} + i\frac{\gamma-\kappa}{2}\right)^2 + 4g^2}\right]. \tag{13}$$

On the other hand, Supplementary Eq. (11) can also be solved analytically for the limiting case where the losses κ and γ are negligible compared to g. Under this condition, the polaritonic frequencies are

$$\omega_{\pm}^{(j)} = \frac{1}{\sqrt{2}}\sqrt{(\omega_{\rm cav}^{(j)})^2 + \omega_{\rm TO}^2 + 4g^2 \pm \sqrt{\left((\omega_{\rm cav}^{(j)})^2 + \omega_{\rm TO}^2 + 4g^2\right)^2 - 4(\omega_{\rm cav}^{(j)})^2\omega_{\rm TO}^2}}. \tag{14}$$

To calculate the coupling strength g, we first extract the polaritonic frequencies $\omega_{\pm}^{(j)}$ from the transfer-matrix simulations (Supplementary Note 2.1). For this calculation, we choose a thickness $L_{\rm cav}$ so that the cavity mode is resonant with the TO phonon, $\omega_{\rm cav}^{(j)} = \omega_{\rm TO}$. Then, we obtain the value for g that minimizes the difference between the two frequencies obtained from this simulation and from the analytical solution. For the latter, we use either Supplementary Eq. (13) or Supplementary

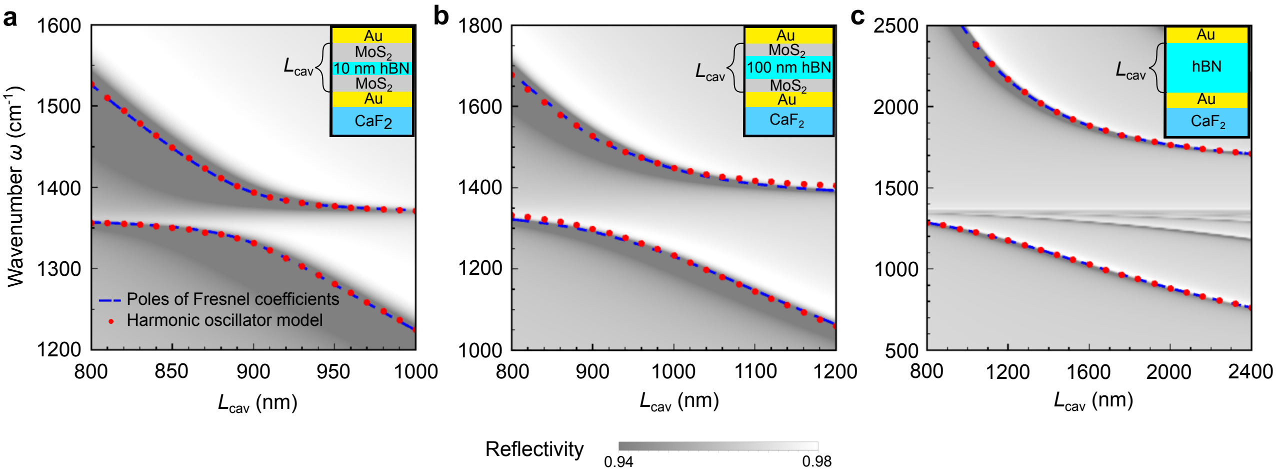

Supplementary Figure 3: Frequency of the polaritonic states formed by the TO phonon and the first cavity mode, for a cavity (a) filled with a 10 nm layer of hBN, (b) filled with a 100 nm layer of hBN and (c) fully filled with hBN. The hBN layer in (a) and (b) is situated in the middle of the cavity. The colormap shows the calculated reflectivity spectra as a function of the incident wavenumber ω and the total thickness L_{cav}. Red dots show the polariton frequencies given by the harmonic oscillator model for (a) $g = 34$ cm^{-1}, (b) $g = 105$ cm^{-1} and (c) $g = 428$ cm^{-1}, and the blue dashed lines show the frequencies extracted from the poles of the Fresnel coefficients.

Eq. (14), depending on the coupling regime (we have verified that the results from these approximate equations are a very good approximation of the exact Supplementary Eq. (11) in their corresponding regimes of application). We follow this procedure to calculate all the values of g given in the main text, and in particular for the analysis of the three cavities in Figs. 3 and 5 of the main text and in Supplementary Fig. 4. The coupling strength that we obtain for the first cavity mode is $g = 34$ cm^{-1} for the cavity embedding a 10 nm-thick hBN layer, $g = 105$ cm^{-1} for the 100 nm-thick layer and $g = 428$ cm^{-1} for the cavity fully filled by hBN.

Further, although we calculated g for the particular case where the cavity is tuned to the TO phonon, we show in Supplementary Fig. 3 that these values of g allow to fully describe the evolution of the polaritonic frequencies as the cavity is detuned. Specifically, Supplementary Fig. 3 shows the polaritonic frequencies obtained from the harmonic oscillator model with (a) $g = 34$ cm^{-1}, (b) $g = 105$ cm^{-1} and (c) $g = 428$ cm^{-1} (red dots) compared with those given by the poles of the total reflection and transmission coefficients for (a) a cavity with a 10 nm-thick hBN layer, (b) a cavity with a 100 nm-thick hBN layer, and (c) a fully filled cavity (dashed blue lines), as a function of the total cavity thickness L_{cav}. These values are superimposed to the colormap of the calculated reflectivity of the cavity. The frequencies obtained from these two methods are almost identical for all L_{cav} and for the three cavities considered.

Interestingly, Supplementary Eq. (14) coincides with the results of the Hopfield Hamiltonian $\hat{H}_{\text{Hop}}$ [12]. In this alternative approach, light and matter excitations are also represented by harmonic oscillators which interact with coupling strength g, but the behaviour of the system is described by the Hamiltonian

$$\hat{H}_{\text{Hop}} = \hbar\omega_{\text{cav}}^{(j)}\hat{a}^\dagger\hat{a} + \hbar\omega_{\text{TO}}\hat{b}^\dagger\hat{b} - i\hbar g\sqrt{\frac{\omega_{\text{TO}}}{\omega_{\text{cav}}^{(j)}}}(\hat{a}+\hat{a}^\dagger)(\hat{b}-\hat{b}^\dagger) + \hbar\frac{g^2}{\omega_{\text{cav}}^{(j)}}(\hat{a}+\hat{a}^\dagger)^2, \quad (15)$$

where $\hat{a}$ and $\hat{a}^\dagger$ are the annihilation and creation operator of the jth cavity mode, respectively, while $\hat{b}$

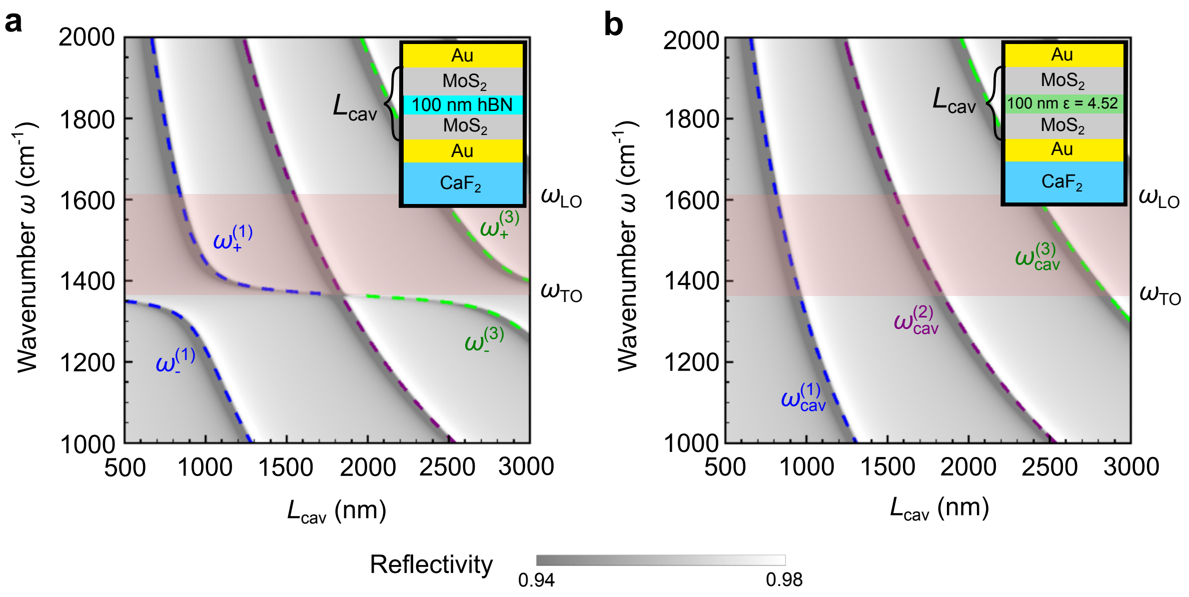

Supplementary Figure 4: Theoretical calculations of the reflectivity spectra of a cavity embedding in the middle of this cavity a 100 nm layer of a) hBN and b) a material with the high-frequency permittivity of hBN, $\varepsilon_{\mathrm{hBN},\infty}$. This layer is situated between two identical MoS_2 slabs. The reflectivity is calculated with transfer-matrix simulations, as a function of the wavenumber of the incident light and the total thickness of the cavity L_{cav}. The shadowed area highlights the Reststrahlen band of the in-plane phonon, delimited by the ω_{LO} and ω_{TO} wavenumbers. The dashed lines represent the wavenumbers of the polariton modes $\omega_+^{(j)}$ and $\omega_-^{(j)}$ in (a) and the bare cavity modes $\omega_{\mathrm{cav}}^{(j)}$ in (b), as obtained from the poles of the reflection and transmission coefficients.

and $\hat{b}^\dagger$ are the corresponding operators for the TO phonon. Apart from the interaction term proportional to g, this Hamiltonian also includes the diamagnetic term, which is proportional to g^2 [13, 14]. Due to the diamagnetic and counter-rotating terms of $\hat{H}_{\mathrm{Hop}}$, the energy of the ground state is shifted from zero. The shift of the ground state is not described by the classical harmonic oscillator model, but in this study we are interested in the transition energies between the excited states and the ground state. The result obtained for these transition energies using the Hopfield Hamiltonian is equal to the polaritonic energies obtained from Supplementary Eq. (14). Thus, the classical harmonic oscillator model is able to describe the transition energies also in the ultrastrong coupling regime [9].

Last, we note that the harmonic oscillator model presented here treats each cavity mode of order j independently, i.e. that different cavity modes do not interact with each other and thus the coupling between hBN and each cavity mode is described by a separate Supplementary Eq. (9). For the cavities that we analyze in this work, which are designed so that the TO phonon interacts mostly with the first cavity mode, the results in Supplementary Fig. 3 show that this approach is appropriate. However, we have found that for thicker cavities where higher-order modes are tuned to the TO phonon, the harmonic oscillator does not always describe well the position of the polaritonic frequencies as obtained from the transfer-matrix simulations. We believe that solving this discrepancy requires to include in the harmonic oscillator model the possibility of coupling different modes with each other, for instance following an approach based on quasinormal modes [15, 16] (a similar reason may be behind the small discrepancy between the two values of the coupling strength that are obtained in Supplementary Fig. 8 for the 3rd cavity mode, as discussed in Supplementary Note 6). However, the results of the cavities analyzed in this work would not be significantly affected by future analysis of this type.

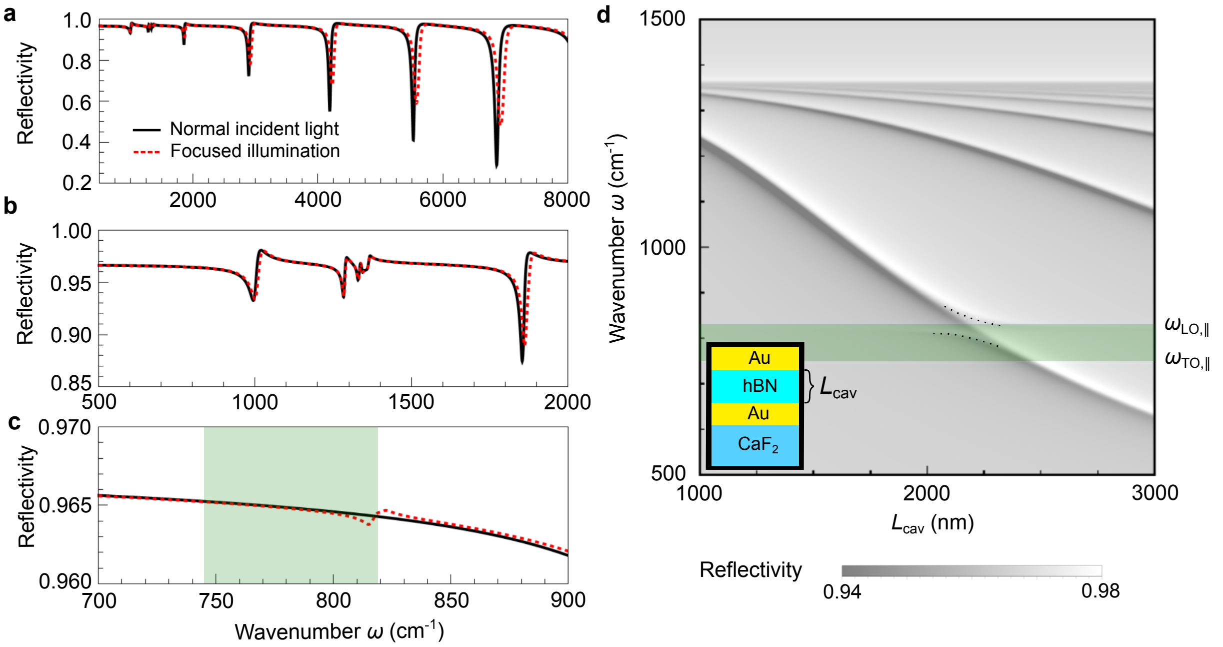

Supplementary Figure 5: Reflectivity spectra calculated for cavities fully filled with hBN under focused illumination. a-c) Comparison between the spectra of a cavity with thickness $L_{\rm cav} = 1665$ nm obtained for normal incident light (black solid lines) and focused illumination (red dashed lines). (b) and (c) correspond to zooms of different regions of the spectra in (a). d) Reflectivity spectra under focused illumination as a function of the total cavity thickness $L_{\rm cav}$ and wavenumber ω of the incident light. Dots are a guide to the peak positions in the region near the anticrossing that occurs due to the coupling of the cavity mode of order 1 and the out-of-plane phonons. The green shadowed area in (c) and (d) represents the Reststrahlen band of the out-of-plane phonon, limited by frequencies $\omega_{\rm TO,\|}$ and $\omega_{\rm LO,\|}$.

3 Reflectivity spectra of the cavity embedding a 100 nm-thick hBN layer

In the main text, we show the reflectivity spectra of a cavity embedding a 10 nm-thick hBN layer (Fig. 3) and a cavity fully filled by hBN (Fig. 5), as a function of the total cavity thickness $L_{\rm cav}$. To complement these results, we show in Supplementary Fig. 4a the reflectivity spectra of the cavity embedding a 100 nm-thick hBN layer. For reference, in Supplementary Fig. 4b we show a calculation of a bare cavity where the 100 nm layer is formed by a material with constant permittivity $\varepsilon_{\rm hBN,\infty}$. The dashed lines in Supplementary Fig. 4a indicate the polaritonic frequencies $\omega_-^{(j)}$ and $\omega_+^{(j)}$ that result from the coupling between the TO phonon and the bare-cavity mode of order j. The dashed lines in Supplementary Fig. 4b correspond to the bare cavity mode frequencies $\omega_{\rm cav}^{(j)}$. As expected, we observe that the anticrossing between the polaritonic frequencies for the 100 nm-thick hBN layer is considerably smaller than for the fully filled cavity, but much larger than for the cavity with a 10 nm layer.

4 Reflectivity of the system under focused illumination

In the transfer-matrix calculations of the main text, we assume that the propagation direction of the incident light is normal to the surface of the mirrors and the substrate (z direction). However, the experiments have been performed using a microscope with focused illumination. In this Supplementary

Note, we discuss the effect of this focusing on the reflectivity spectra of the system. We show that this effect is small and that the main difference with respect to the spectra under normal incident light is the possibility to observe the coupling with the out-of-plane phonons of hBN, instead of just with the in-plane phonons (see Supplementary Note 1 for a discussion of these two types of phonons).

The total reflectivity $\mathcal{R}_{\rm foc}$ for focused illumination is obtained by decomposing the incident light as an integral over planewaves incident at different angles. We then obtain (for more details, see Chapter 3.9 in Supplementary Ref. [17]):

$$\mathcal{R}_{\rm foc} = \frac{I_{\rm ref}}{I_{\rm in}} = \frac{\int_{\sin\theta_{\rm min}}^{\sin\theta_{\rm max}} \frac{|r^s_{\rm total}(\xi)|^2 + |r^p_{\rm total}(\xi)|^2}{2}\, \xi \, d\xi}{\int_{\sin\theta_{\rm min}}^{\sin\theta_{\rm max}} \xi \, d\xi}, \tag{16}$$

where $I_{\rm in}$ and $I_{\rm ref}$ correspond to the intensity of the incident and reflected focused beam, respectively, $\xi = \frac{\sqrt{k_x^2+k_y^2}}{k_0} = \frac{k_\parallel}{k_0}$ is the normalized parallel wavevector and $r^{s(p)}_{\rm total}$ is the ξ-dependent reflection coefficient of the full system for a $s(p)$-polarized planewave, which is discussed thoroughly in Supplementary Note 2.1. The upper limit of the integral is given by the numerical aperture of the microscope used to focus light, NA $= \sin\theta_{\rm max}$. In our case, we have NA $= 0.4$, and thus, $\theta_{\rm max} \approx 23.5°$. Furthermore, we set $\theta_{\rm min} = 10°$ for the lower bound of the integral, because the microscope used in the experiments obstructs the propagation of the central part of the light incoming to the focusing lens, which eliminates the contribution from the small-angle components.

We have verified that, for cavities embedding a 10 nm layer of hBN, the results of the transfer-matrix simulations using focused illumination are nearly identical compared to the spectra for normal incidence. Crucially, light travels at considerably smaller angles inside this cavity than in free space, due to the high permittivity of $\mathrm{MoS_2}$, $\varepsilon_{\rm MoS2}$, which fills most of the cavity for such a thin hBN layer. In particular, the angles inside $\mathrm{MoS_2}$ range from 2.6° to 5.9°, and thus the difference with the case of normal incidence is very small.

We next consider a fully filled hBN cavity of thickness $L_{\rm cav} = 1665$ nm (whose first cavity mode is resonant with the TO phonon frequency) and show in Supplementary Fig. 5(a-c) the reflectivity spectra under focused (red dashed line) and normal-incidence illumination (black solid line), both calculated by transfer-matrix simulations. The effect of using focused light becomes larger for the cavity fully filled by hBN, as the high-frequency permittivity of hBN $\varepsilon_{\rm hBN,\infty}$ is smaller than $\varepsilon_{\rm MoS2}$, and thus the angle of light propagation inside the cavity can be larger. A small but appreciable difference between normal incident light and focused illumination is observed for high wavenumbers, as shown in panel a. The reflectivity dips obtained under focused illumination are displaced towards larger wavenumbers compared to the normal-incidence spectra, because the frequencies of the Fabry-Pérot cavity modes depend on the angle of propagation. Furthermore, the widths of the dips are larger for focused illumination because they are the result of the sum of different contributions, each corresponding to a different angle and thus resonant at a slightly different wavenumber (Supplementary Eq. (16)). However, these differences remain small, and the agreement between the two spectra improves further for frequencies close to $\omega_{\rm TO}$ (see zoom in panel b), which is the main region of interest in this work. Thus, the use of normal incidence in our calculations of the other sections is justified.

Possibly the most interesting feature of the results obtained under focused illumination is observed in Supplementary Fig. 5c, which shows the reflectivity spectra at low wavenumbers (near the frequencies of the out-of-plane phonons). An additional reflectivity dip for focused illumination (red

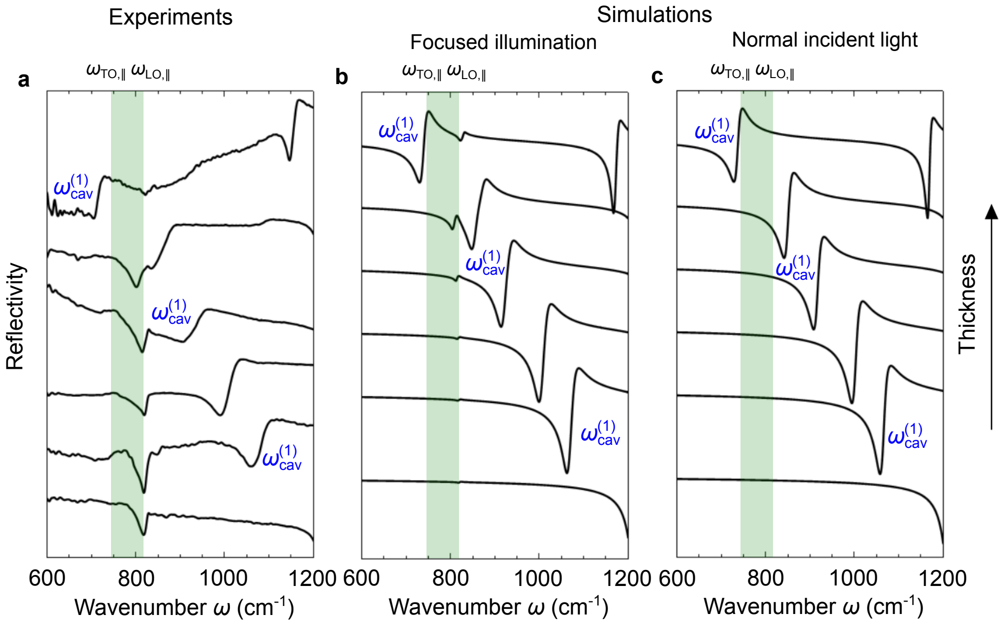

Supplementary Figure 6: Reflectivity spectra of the fully filled hBN cavities at low wavenumbers, obtained from a) the experiments; b) transfer-matrix simulations under focused illumination; and c) transfer-matrix simulations with a planewave incoming in the normal direction. The Reststrahlen band of the out-of-plane phonon is highlighted by the green area. The (a) nominal and (b,c) simulated thicknesses L_{cav} of the cavities (from bottom to top) are: 1080 nm, 1500 nm, 1665 nm, 1900 nm, 2100 nm and 2500 nm. Panel a is a zoom of Fig. 5c of the main text in the region of low wavenumbers.

dashed line) appears compared to the normal-incidence spectra (black solid line), which is due to the anisotropy of hBN. For normal incidence, the illumination only couples with the in-plane transverse optical phonon, which is polarized in the parallel direction to all planar interfaces. On the other hand, for focused illumination it becomes possible to excite the phonons that are polarized in the out-of-plane direction and that are found at significantly lower frequencies ($\omega_{\mathrm{TO},\|} = 746\ \mathrm{cm}^{-1}$ and $\omega_{\mathrm{LO},\|} = 819\ \mathrm{cm}^{-1}$, the Reststrahlen band of the out-of-plane phonons limited by these frequencies is highlighted with the green area in Supplementary Fig. 5). These phonons strongly affect the z component of the permittivity tensor, and thus they can couple with the electric field components in the z direction of the focused light, which explains the extra dip.

For a more detailed analysis of this coupling, we show in Supplementary Fig. 5d the calculated reflectivity spectra under focused illumination as a function of the total cavity thickness L_{cav}. When the frequency of the bare-cavity mode gets close to the Reststrahlen band of the out-of-plane phonon, an anticrossing characterized by a relatively small splitting is observed, as indicated by the dots as a guide. Hence, the use of a focused beam makes possible to observe the coupling of the cavity modes with in-plane and out-of-plane phonons.

The coupling with the out-of-plane phonons has also been observed in the experimental spectra of the fully filled hBN cavities. Supplementary Figure 6a shows the measured reflectivity spectra for all six cavities. This figure corresponds to a zoom of Fig. 5c in the main text on the region near the Reststrahlen band associated with the out-of-plane phonons (green area). We observe a dip labelled by $\omega_{\mathrm{cav}}^{(1)}$ that changes strongly with the thickness of the cavity and it is mostly associated to the bare-cavity mode. The other dip is close to $\omega_{\mathrm{LO},\|}$, and its frequency is also indicated by the yellow dots in Fig. 5a of the main text. In order to confirm the nature of the dips at frequency $\approx \omega_{\mathrm{LO},\|}$,

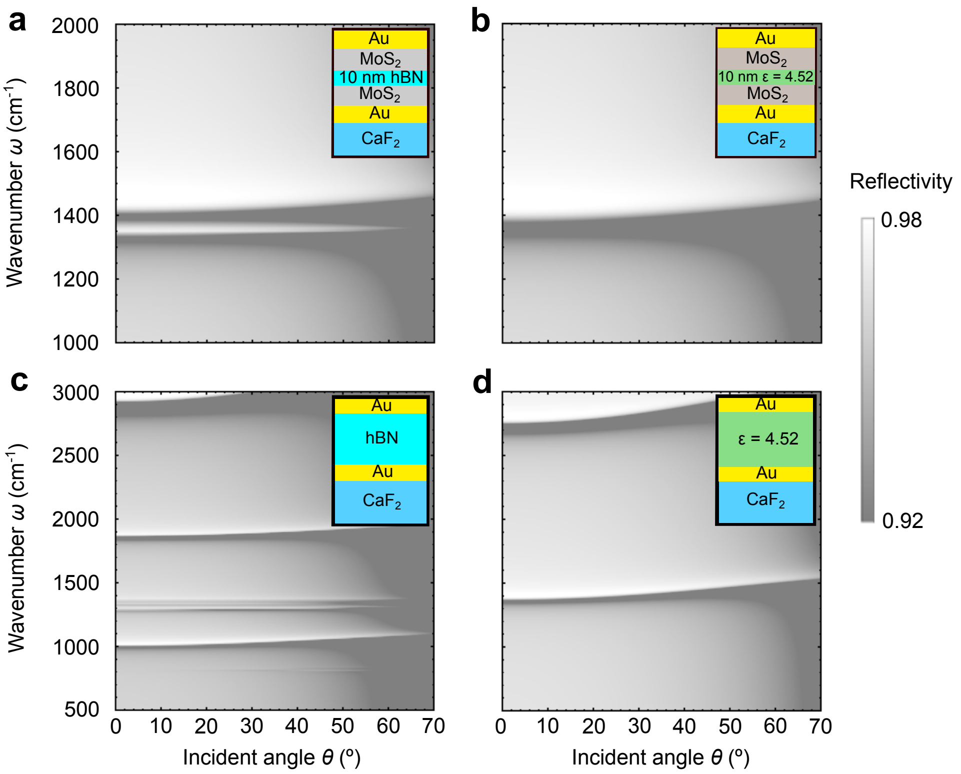

Supplementary Figure 7: Reflectivity spectra calculated for p-polarized light as a function of the incident angle θ and wavenumber ω for a) a MoS_2/hBN/MoS_2 cavity with thicknesses 510 nm/10 nm/370 nm, b) a bare cavity with the same thicknesses as in (a), c) a fully filled cavity with thickness $L_{\rm cav}$ = 1665 nm, d) a bare cavity with the same thickness as in (c). The structures are shown schematically in the insets.

we also show in Supplementary Fig. 6 the results from the transfer-matrix simulations under focused illumination (panel b) and normal incident light (panel c). For panel c, we only see the dip associated to the bare-cavity mode at frequency $\omega_{\rm cav}^{(1)}$. On the other hand, once we consider focused illumination, we can observe the second feature near $\omega_{\rm LO,||}$ that was identified in the experiments. We thus confirm that the extra peak only appears in the calculations when the polarization has a nonzero z component and thus the illumination couples also with the out-of-plane phonons. However, we notice that the size of the experimental dips is significantly larger than the simulated ones, and we attribute this discrepancy to experimental imperfections such as rugosities (which can scatter light at high angles) or non-perfect planarity of the fabricated cavities. Despite this difference, the good agreement on the spectral positions of the dips indicates that they are indeed a result of the coupling between the first cavity mode and the out-of-plane phonons.

5 Angle dependence of the reflectivity spectra

We show in Supplementary Fig. 7 the reflectivity spectra of hBN cavities under p-polarized light, as a function of the angle θ of the incident illumination with respect to the normal axis. Panel a corresponds to the cavity with thicknesses 510 nm/10 nm/370 nm of the MoS_2/hBN/MoS_2 layers (see inset), which is analyzed in Fig. 2 in the main text. The results depend very weakly on the angle θ of the incident illumination because the corresponding angle of the refracted beam inside the cavity is much smaller than θ (due to the high permittivities of the materials filling the cavity). For reference,

we show in Supplementary Fig. 7b the spectra of the bare cavity with the same thickness, which shows the same weak dependence on the angle.

Further, we show the spectra of a cavity of thickness $L_{\text{cav}} = 1665$ nm fully filled with hBN (Supplementary Fig. 7c) and with a material of permittivity $\varepsilon_{\text{hBN},\infty} = 4.52$ (Supplementary Fig. 7d). We observe again that the influence of the angle on the reflectivity is small. The main effect of increasing θ is the emergence in Supplementary Fig. 7c of new faint reflectivity dips close to the frequency of the out-of-plane hBN phonon $\omega_{\text{LO},\|} = 819$ cm^{-1}. This additional dip indicates that for oblique incident angles it is possible to observe the coupling of the cavity with the out-of-plane phonon. Last, we have checked that the reflectivity spectra under s-polarized light contains almost the same dips as in Supplementary Fig. 7. The main difference is that in this case there is no dip near $\omega_{\text{LO},\|}$ (i.e. it is not possible to couple with the out-of-plane phonon), because the electric field is polarized in the in-plane direction.

6 Analytical expression for the coupling strength

6.1 Coupling strength of a TO phonon with a Fabry-Pérot cavity mode

In Supplementary Note 2.3, we describe the method that we follow in the main text to calculate the coupling strength g for hBN cavities, by extracting the polaritonic frequencies from the poles of the reflection and transmission coefficients and fitting these frequencies with a model of coupled harmonic oscillators. In this note, we present an alternative approach based on the microscopic interaction between the phononic material and the modes of a Fabry-Pérot cavity, which allows for deriving an approximate analytical expression for g, and can give additional physical insight about the coupling. An analogous approach has been followed in Supplementary Ref. [18] to model the coupling strength between molecules and guided nanowire modes.

In this derivation, we consider a simplified picture where we model each unit cell of hBN as a harmonic oscillator characterized by a transition dipole moment $\mathbf{d}_i$. The dipole is induced by the vibrations of the atoms. From this perspective, the material is a collection of N_{cell} dipoles interacting with the cavity mode. Due to the homogeneity of the material, the transition dipole moments of all unit cells are the same: $\mathbf{d}_i = \mathbf{d}$. All dipoles are oriented parallel to the electric field, and in order to deduce the module $d_i = |\mathbf{d}_i|$, we first consider the electric susceptibility of the material, which for a polar material such as hBN is

$$\chi(\omega) = \varepsilon_\infty \frac{\omega_{\text{LO}}^2 - \omega_{\text{TO}}^2}{\omega_{\text{TO}}^2 - \omega^2 - i\omega\gamma}. \tag{17}$$

ω_{TO} and ω_{LO} are the frequencies of the transverse optical and the longitudinal optical phonon, respectively, γ is the damping frequency and ε_∞ is the high-frequency permittivity. From this expression, we can directly obtain the polarizability α_i of each dipole induced in a unit cell of volume V_{cell}. We consider that the polarization density $\mathbf{P}$ is related to the electric field $\mathbf{E}$ as $\mathbf{P} = \frac{\alpha_i}{V_{\text{cell}}}\mathbf{E} = \varepsilon_0\chi\mathbf{E}$, and we therefore obtain

$$\alpha_i(\omega) = \varepsilon_0 V_{\text{cell}} \chi(\omega), \tag{18}$$

where ε_0 is the vacuum permittivity. We can now relate the transition dipole moment d_i and the classical polarizability by [17]

$$\alpha_i(\omega) = \frac{2\omega_{\text{TO}}}{\hbar} \frac{d_i^2}{\omega_{\text{TO}}^2 - \omega^2 - i\omega\gamma}, \tag{19}$$

and, from Supplementary Eqs. (17)–(19):

$$d_i = \sqrt{\frac{\hbar}{2\omega_{\rm TO}} V_{\rm cell}\varepsilon_0\varepsilon_\infty(\omega_{\rm LO}^2 - \omega_{\rm TO}^2)}. \tag{20}$$

This expression allows to characterize the coupling strength g_i between the dipole associated to a particular unit cell and the cavity mode with quantized electric field $\hat{\mathbf{E}}(\mathbf{r})$. This electric field corresponds to a bare cavity where the hBN is substituted by a material of frequency-independent, real permittivity ε_∞ (i.e., without considering the resonant polarizability of the unit cells). In this derivation, we consider normal incidence of the excitation light. For the quantization of the field, we further assume that the mirrors of the cavity are perfect, so that the electric and magnetic energy of the modes are equal and the fields do not penetrate into the mirrors. The electric field of the Fabry-Pérot mode of order j resonant at frequency $\omega_{\rm cav}^{(j)}$ is quantized as [19]

$$\hat{\mathbf{E}} = \mathbf{E}(\hat{a} + \hat{a}^\dagger) = \sqrt{\frac{\hbar\omega_{\rm cav}^{(j)}}{2\varepsilon_0 V_{\rm eff}}}\rho(z)(\hat{a} + \hat{a}^\dagger)\hat{\mathbf{u}}_x. \tag{21}$$

$\hat{a}$ and $\hat{a}^\dagger$ are the annihilation and creation operators of the cavity mode, respectively. The fields only vary along the z direction normal to the flat interfaces of the system, as given by the field profile $\rho(z)$ (normalized so that $\rho(z) = 1$ in the position where the field amplitude is maximum). The electric field is assumed to be polarized along the x axis, with unit vector $\hat{\mathbf{u}}_x$. $V_{\rm eff} = S\int \varepsilon(z)|\rho(z)|^2 dz$ is the effective volume of the field, where S is the effective surface of the cavity and $\varepsilon(z)$ indicates the spatial distribution of the permittivities of the system. The value of the electric field $\mathbf{E}(\mathbf{r})$ of a Fabry-Pérot mode is related to the coupling strength g_i between that mode and the dipole excited in the unit cell at position $\mathbf{r}_i$, according to

$$\hbar g_i = -\mathbf{d}_i \cdot \mathbf{E}(\mathbf{r}_i). \tag{22}$$

Taking into account that the dipole moments and the electric field are parallel, we write this coupling strength as

$$g_i = -\sqrt{\frac{\omega_{\rm cav}^{(j)}}{\omega_{\rm TO}} \frac{\omega_{\rm LO}^2 - \omega_{\rm TO}^2}{4} \frac{V_{\rm cell}\varepsilon_\infty \rho(z_i)^2}{S\int \varepsilon(z)|\rho(z)|^2 dz}}. \tag{23}$$

We next consider the Hamiltonian describing the interaction of the cavity mode with the ensemble of dipoles associated with all the unit cells in the phononic material

$$\hat{H} = \hbar\omega_{\rm cav}^{(j)}\hat{a}^\dagger\hat{a} + \hbar\omega_{\rm TO}\sum_{i=1}^{N_{\rm cell}}\hat{b}_i^\dagger\hat{b}_i + \hbar(\hat{a} + \hat{a}^\dagger)\sum_{i=1}^{N_{\rm cell}}(g_i\hat{b}_i + g_i^*\hat{b}_i^\dagger). \tag{24}$$

where $\hat{b}_i$ and $\hat{b}_i^\dagger$ are the annihilation and creation operator of the harmonic oscillator that represents each vibrational dipole, respectively. The next step is to write the Hamiltonian of the system in terms of the annihilation $\hat{b}_{\rm c}$ and creation $\hat{b}_{\rm c}^\dagger$ operators of the bright collective excitation that represents the TO phonon,

$$\hat{H} = \hbar\omega_{\rm cav}^{(j)}\hat{a}^\dagger\hat{a} + \hbar\omega_{\rm TO}\hat{b}_{\rm c}^\dagger\hat{b}_{\rm c} + \hbar\omega_{TO}\sum_{i=1}^{N_{\rm cell}-1}\hat{c}_i^\dagger\hat{c}_i + \hbar g(\hat{a} + \hat{a}^\dagger)(\hat{b}_{\rm c} + \hat{b}_{\rm c}^\dagger), \tag{25}$$

where we have also added the annihilation $\hat{c}_i$ and creation $\hat{c}_i^\dagger$ operators of the $N_{\rm cell} - 1$ dark collective modes that are uncoupled to the cavity mode. Supplementary Eq. (25) can be derived from

Supplementary Eq. (24) by using the Dicke transformation

$$\sum_{i=1}^{N_{\text{cell}}} \hat{b}_i^\dagger \hat{b}_i = \hat{b}_{\text{c}}^\dagger \hat{b}_{\text{c}} + \sum_{i=1}^{N_{\text{cell}}-1} \hat{c}_i^\dagger \hat{c}_i, \qquad \sum_{i=1}^{N_{\text{cell}}} g_i \hat{b}_i = g \hat{b}_{\text{c}}, \qquad \sum_{i=1}^{N_{\text{cell}}} g_i^* \hat{b}_i^\dagger = g^* \hat{b}_{\text{c}}^\dagger, \tag{26}$$

where g takes the value [20]

$$g = \sqrt{\sum_{i=1}^{N_{\text{cell}}} |g_i|^2} \tag{27}$$

so that the commutation relation $[\hat{b}_{\text{c}}, \hat{b}_{\text{c}}^\dagger] = 1$ is fulfilled. The operators of the dark modes fulfill the commutation relations $[\hat{c}_i, \hat{c}_j^\dagger] = \delta_{ij}$ and $[\hat{b}_c, \hat{c}_i^\dagger] = 0$. Thus, Supplementary Eq. (27) gives the coupling strength between the cavity mode and the collective excitation (the TO phonon) in terms of the coupling strengths g_i associated to the individual dipoles of each unit cell. g is evaluated by replacing Supplementary Eq. (23) into Supplementary Eq. (27), and transforming the sum into an integral (since the dipoles are distributed in a continuous way). The result is

$$\begin{aligned} |g|^2 &= \sum_{i=1}^{N_{\text{cell}}} \frac{\omega_{\text{cav}}^{(j)}}{\omega_{\text{TO}}} \frac{\omega_{\text{LO}}^2 - \omega_{\text{TO}}^2}{4} \frac{V_{\text{cell}} \varepsilon_\infty |\rho(z_i)|^2}{S \int \varepsilon(z') |\rho(z')|^2 dz'} = \int \frac{\omega_{\text{cav}}^{(j)}}{\omega_{\text{TO}}} \frac{\omega_{\text{LO}}^2 - \omega_{\text{TO}}^2}{4} \frac{V_{\text{cell}} \varepsilon_\infty |\rho(z)|^2}{S \int \varepsilon(z') |\rho(z')|^2 dz'} \frac{dN_{\text{cell}}}{dV} dV \\ &= \frac{\omega_{\text{cav}}^{(j)}}{\omega_{\text{TO}}} \frac{\omega_{\text{LO}}^2 - \omega_{\text{TO}}^2}{4} \frac{\int \varepsilon_\infty |\rho(z)|^2 dz}{\int \varepsilon(z') |\rho(z')|^2 dz'}, \end{aligned} \tag{28}$$

where in the last step we have taken into account that the density of dipoles is $\frac{dN_{\text{cell}}}{dV} = \frac{1}{V_{\text{cell}}}$ and the effective surface S of the mode is eliminated after the integration in the $x - y$ plane. The integral of the denominator in Supplementary Eq. (28) is evaluated in the entire cavity, while the integral of the numerator is bounded to the region of the phononic material. Therefore, in this subsection we have obtained an expression that allows to calculate the coupling strength between the TO phonon of a polar material and a Fabry-Pérot mode of a cavity with an arbitrary spatial distribution of the permittivity $\varepsilon(z)$ (and thus arbitrary field distribution $\rho(z)$).

6.2 Application to the hBN microcavities

In this subsection, we focus on the particular system that we analyze in the main text, a cavity of thickness L_{cav} with MoS_2 as a spacer and containing a layer of hBN (details can be found in Supplementary Note 1). First, we need to calculate the field profile $\rho(z)$ of the modes of the bare cavity, in order to evaluate the coupling strength g through Supplementary Eq. (28). Labelling the positions of the MoS_2-hBN interfaces as L_1 and L_2 (indicated in Supplementary Fig. 1), the spatial distribution of the permittivity $\varepsilon(z)$ of the bare cavity is described by the function

$$\varepsilon(z) = \begin{cases} \varepsilon_{\text{MoS}_2}, & 0 < z < L_1 \\ \varepsilon_{\text{hBN},\infty}, & L_1 < z < L_2 \\ \varepsilon_{\text{MoS}_2}, & L_2 < z < L_{\text{cav}} \end{cases}. \tag{29}$$

In each interval with constant permittivity ε_i, the field profile $\rho(z)$ satisfies the Helmholtz equation

$$\frac{d^2\rho(z)}{dz^2} + \frac{\varepsilon_i (\omega_{\text{cav}}^{(j)})^2}{c^2} \rho(z) = 0, \tag{30}$$

where c is the speed of light in vacuum. Further, since the cavity is assumed to be bounded by perfect mirrors, the electric field vanishes at both ends: $\rho(0) = \rho(L_{\rm cav}) = 0$. In order to verify the boundary conditions, $\rho(z)$ needs to be continuous and differentiable in all interfaces, which leads to the solution

$$\rho(z) = \begin{cases} \mathcal{A}\frac{\sin\left(\frac{\omega_{\rm cav}^{(j)}}{c}\sqrt{\varepsilon_{\rm hBN,\infty}}L_1+\phi^{(j)}\right)}{\sin\left(\frac{\omega_{\rm cav}^{(j)}}{c}\sqrt{\varepsilon_{\rm MoS_2}}L_1\right)}\sin\left(\frac{\omega_{\rm cav}^{(j)}}{c}\sqrt{\varepsilon_{\rm MoS_2}}z\right) & 0 < z < L_1 \\ \mathcal{A}\sin\left(\frac{\omega_{\rm cav}^{(j)}}{c}\sqrt{\varepsilon_{\rm hBN,\infty}}z + \phi^{(j)}\right) & L_1 < z < L_2 \\ \mathcal{A}\frac{\sin\left(\frac{\omega_{\rm cav}^{(j)}}{c}\sqrt{\varepsilon_{\rm hBN,\infty}}L_1+\phi^{(j)}\right)}{\sin\left(\frac{\omega_{\rm cav}^{(j)}}{c}\sqrt{\varepsilon_{\rm MoS_2}}L_1\right)}\sin\left(\frac{\omega_{\rm cav}^{(j)}}{c}\sqrt{\varepsilon_{\rm MoS_2}}(z-L_{\rm cav})\right), & L_2 < z < L_{\rm cav} \end{cases}. \quad (31)$$

$\mathcal{A}$ is the normalization constant chosen so that the maximum of the field profile is $\rho(z) = 1$, while $\omega_{\rm cav}^{(j)}$ and $\phi^{(j)}$ are the $j^{\rm th}$ solution of the system of equations

$$\sqrt{\varepsilon_{\rm MoS_2}}\cot\left(\frac{\omega_{\rm cav}^{(j)}}{c}\sqrt{\varepsilon_{\rm MoS_2}}L_1\right) = \sqrt{\varepsilon_{\rm hBN,\infty}}\cot\left(\frac{\omega_{\rm cav}^{(j)}}{c}\sqrt{\varepsilon_{\rm hBN,\infty}}L_1 + \phi^{(j)}\right) \quad (32a)$$

$$\sqrt{\varepsilon_{\rm MoS_2}}\cot\left(\frac{\omega_{\rm cav}^{(j)}}{c}\sqrt{\varepsilon_{\rm MoS_2}}(L_2 - L_{\rm cav})\right) = \sqrt{\varepsilon_{\rm hBN,\infty}}\cot\left(\frac{\omega_{\rm cav}^{(j)}}{c}\sqrt{\varepsilon_{\rm hBN,\infty}}L_2 + \phi^{(j)}\right). \quad (32b)$$

We can obtain g by inserting Supplementary Eq. (31) into Supplementary Eq. (28), with the integral extending over the phononic material (between L_1 and L_2), i.e. we evaluate

$$|g|^2 = \frac{\omega_{\rm LO}^2 - \omega_{\rm TO}^2}{4}\frac{\int_{L_1}^{L_2}\varepsilon_{\rm hBN,\infty}|\rho(z)|^2 dz}{\int_0^{L_{\rm cav}}\varepsilon(z')|\rho(z')|^2 dz'}. \quad (33)$$

In this calculation, we always assume that the cavity mode of order j is resonant with the TO phonon, $\omega_{\rm TO} = \omega_{\rm cav}^{(j)}$, so that the cavity thickness $L_{\rm cav}$ is different for each order and the permittivity of MoS_2 is evaluated at that frequency ($\varepsilon_{\rm MoS_2} \equiv \varepsilon_{\rm MoS_2}(\omega_{\rm TO})$ in this subsection). Adjusting the cavity parameters (specially the thickness of the hBN layer) allows for varying g from 0 to a maximum value $g_{\rm max}$ corresponding to the fully filled cavity that can be evaluated to be $g_{\rm max} = \sqrt{\frac{\omega_{\rm LO}^2-\omega_{\rm TO}^2}{4}}$. Interestingly, we observe that $g_{\rm max}$ only depends on the frequencies of the transverse optical phonon and the longitudinal optical phonon. For hBN, $g_{\rm max} = 428$ cm^{-1}.

Further, we can also obtain a simple analytical expression of g for thin layers of hBN placed in the middle of the cavity and interacting with the first cavity mode. In order to obtain the field profile $\rho(z)$ in this regime, we consider that the thin layer of hBN disturbs very weakly the electric field of the cavity fully filled with MoS_2 and with the same total thickness $L_{\rm cav}$. Under this assumption, we obtain that $\rho(z) \approx \sin\left(\frac{\omega_{\rm cav}^{(1)}}{c}\sqrt{\varepsilon_{\rm MoS_2}}z\right)$, with $\omega_{\rm cav}^{(1)} = \frac{\pi c}{\sqrt{\varepsilon_{\rm MoS_2}}L_{\rm cav}}$. Moreover, since the thin layer is located in the position of the maximum amplitude of the electric field, this amplitude varies slowly inside the hBN layer, and we can assume that the field is constant between L_1 and L_2 ($\rho(z) \approx 1$). By evaluating the integrals in Supplementary Eq. (33) for $L_1 = \frac{L_{\rm cav}}{2} - \frac{L_{\rm hBN}}{2}$ and $L_2 = \frac{L_{\rm cav}}{2} + \frac{L_{\rm hBN}}{2}$, we obtain

$$g \approx \sqrt{\frac{\omega_{\rm LO}^2 - \omega_{\rm TO}^2}{2}\frac{\varepsilon_{\rm hBN,\infty}}{\varepsilon_{\rm MoS_2}}\frac{L_{\rm hBN}}{L_{\rm cav}}} \approx 332\ {\rm cm}^{-1}\sqrt{\frac{L_{\rm hBN}}{L_{\rm cav}}}. \quad (34)$$

This result is consistent with the Dicke model [21, 22] that has been often applied to quantify the coupling strength between a cavity mode and an ensemble of N identical molecules [23–25]. Since in

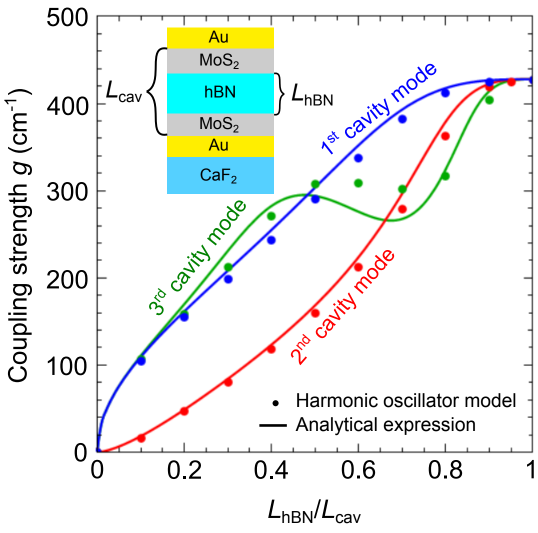

Supplementary Figure 8: Evolution of the coupling strength g as a function of the ratio $\frac{L_{\mathrm{hBN}}}{L_{\mathrm{cav}}}$ (shown in the inset) for the first (blue), the second (red) and the third (green) cavity modes. Solid lines represent the solution of Supplementary Eq. (33), and the dots correspond to the values obtained from the coupled harmonic oscillator model (blue and red dots correspond to the values shown in Fig. 4 of the main text). The cavity thickness L_{cav} is changed so that the cavity is always resonant with the TO phonon frequency.

our case the thickness of the hBN layer L_{hBN} is proportional to the amount of dipoles interacting with the cavity mode, we obtain the same dependency of g on the amount of matter excitations $g \propto \sqrt{N}$ as predicted by Dicke.

We assess in Supplementary Fig. 8 the validity of our analytical model by comparing the result of Supplementary Eq. (33) with the coupling strengths calculated by applying the coupled harmonic oscillator model to the polaritonic frequencies calculated with the transfer-matrix method. For the calculation of the polaritonic frequencies, we always choose the thickness L_{cav} so that the frequency of the cavity mode is resonant with the TO phonon frequency. We show the evolution of the coupling strength g as a function of the filling factor $\frac{L_{\mathrm{hBN}}}{L_{\mathrm{cav}}}$ for the first three cavity modes. Generally, g increases with the filling factor, particularly strongly for thin layers and odd modes, until for all modes it saturates at the same value g_{max} for fully filled cavities. The third mode also shows a non-intuitive tendency of the coupling strength which decreases with an increase of $\frac{L_{\mathrm{hBN}}}{L_{\mathrm{cav}}}$ (in the interval $0.5 \lesssim \frac{L_{\mathrm{hBN}}}{L_{\mathrm{cav}}} \lesssim 0.7$). The reason is that changing L_{hBN} also modifies L_{cav} (to keep the cavity resonant with the TO phonon) and the field distribution inside the cavity, which leads to a complex tendency. Further, for the coupling strength of the first two modes, we observe that the agreement between the analytical expression (solid lines) and the harmonic oscillator model (dots) is very good, with only a slight overestimation by the analytical equation for intermediate values of the filling factor. The agreement becomes worse for the third cavity mode and intermediate filling factors, but it remains generally reasonable. Thus, we confirm that the simple analytical model presented here is able to explain the evolution of the coupling strength with the filling factor, particularly for the first two cavity modes considered in the main text.

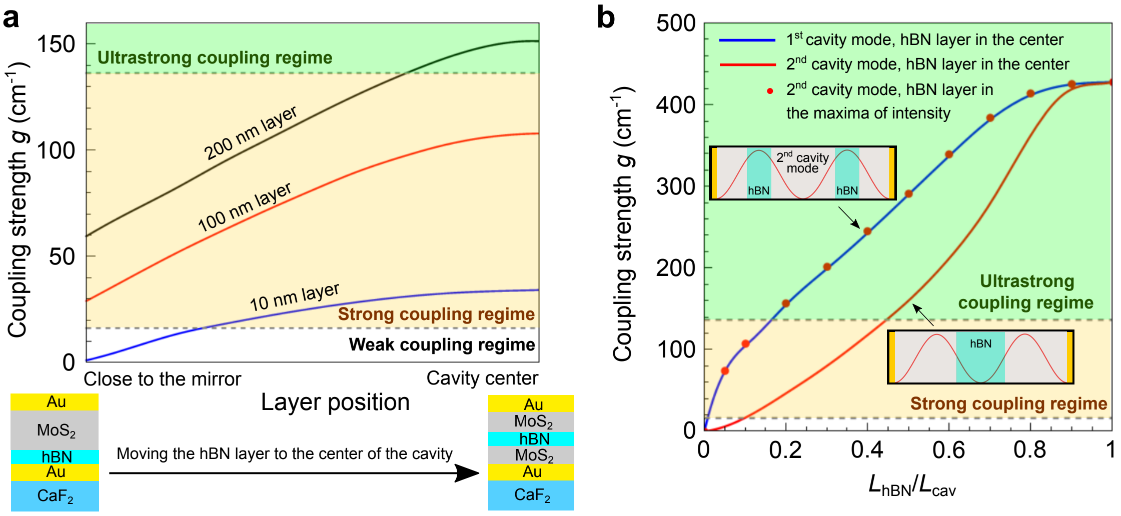

Supplementary Figure 9: (a) Coupling strength g between the TO phonon and the first cavity mode as a function of the hBN layer position, for $L_{\mathrm{hBN}} = 10$ nm (blue line), $L_{\mathrm{hBN}} = 100$ nm (red line) and $L_{\mathrm{hBN}} = 200$ nm (black line). The layer is moved from the position closest to the bottom gold mirror (corresponding to the left of the x axis in the figure, see sketch) to the center of the cavity (right of the x axis). (b) Evolution of the coupling strength g as a function of the filling factor $\frac{L_{\mathrm{hBN}}}{L_{\mathrm{cav}}}$. Blue line: the hBN layer is placed in the middle of the cavity, interacting with the first cavity mode. Red line: the hBN layer is placed in the middle of the cavity, interacting with the second cavity mode. Red dots: the hBN layer is separated into two equally thick hBN layers, which are placed at the intensity maxima of the second cavity mode, interacting with this mode. The insets show the intensity distribution of the second cavity mode interacting with either a single hBN layer of thickness L_{hBN} in the center of the cavity or two hBN layers of thickness $\frac{L_{\mathrm{hBN}}}{2}$. Solid lines correspond to the results shown in Fig. 4 of the main text. The beige area indicates the values of g in the strong coupling regime, while the green area corresponds to the ultrastrong coupling regime.

7 Dependence of the coupling strength on the hBN layer position

We show in Supplementary Fig. 9a the dependence of the coupling strength g on the position of a 10 nm hBN layer (blue line), a 100 nm hBN layer (red line) and a 200 nm hBN layer (black line) placed inside the cavity. The total thickness of the cavity is chosen so that the first cavity mode is resonant with the TO phonon frequency, and MoS_2 is chosen as the spacer material (see sketches). Displacing the 10 nm hBN layer from a position near a mirror (left of the x axis in the figure) to the center of the cavity (right of the axis) induces a transition from the weak to the strong coupling regime. In a similar way, the coupling strength for the cavity containing a 100 nm hBN layer is tuned along a range of values corresponding to the strong coupling regime. Last, for $L_{\mathrm{hBN}} = 200$ nm, the system is tuned from the strong to the ultrastrong coupling regime. Therefore, the results in Supplementary Fig. 9a show that displacing a hBN layer of fixed thickness inside the cavity also makes possible to control the coupling strength [26].

The influence of the position of the hBN layer on the coupling strength is further emphasized in Supplementary Fig. 9b. We plot the coupling strength g as a function of the filling factor $\frac{L_{\mathrm{hBN}}}{L_{\mathrm{cav}}}$ for different situations. We first show that, when the hBN layer is placed in the center of the cavity, the coupling strength with the second cavity mode (red line) is generally much smaller than with the first mode (blue line), because in this position the intensity of the cavity mode vanishes for the former and is maximum for the latter. Further, we consider a situation that maximizes the coupling strength with the second cavity mode (red dots). To obtain these results, we calculate the intensity

distibution associated with the mode and place two identical hBN layers of thickness $\frac{L_{\mathrm{hBN}}}{2}$ centered in the maxima (antinodes). We observe that the evolution of g is almost identical for the first cavity mode with a hBN layer in the center of the cavity and for the second mode with the layers in the positions of maximum intensity of the mode.

Supplementary References

[1] Johnson, P. B. & Christy, R. W. Optical constants of the noble metals. *Phys. Rev. B* **6**, 4370 (1972).

[2] Esteban, R. et al. A classical treatment of optical tunneling in plasmonic gaps: extending the quantum corrected model to practical situations. *Faraday Discuss.* **178**, 151–183 (2015).

[3] Autore, M. et al. Boron nitride nanoresonators for phonon-enhanced molecular vibrational spectroscopy at the strong coupling limit. *Light: Science & Applications* **7**, 17172 (2018).

[4] Menghrajani, K. S., Nash, G. R. & Barnes, W. L. Vibrational strong coupling with surface plasmons and the presence of surface plasmon stop bands. *ACS Photonics* **6**, 2110-2116 (2019).

[5] Passler, N. C. & Paarmann, A. Generalized 4 × 4 matrix formalism for light propagation in anisotropic stratified media: study of surface phonon polaritons in polar dielectric heterostructures. *J. Opt. Soc. Am. B* **34**, 2128–2139 (2017).

[6] Novotny, L. Strong coupling, energy splitting, and level crossings: a classical perspective. *Am. J. Phys.* **78**, 1199 (2010).

[7] Wu, X., Gray, S. K. & Pelton, M. Quantum-dot-induced transparency in a nanoscale plasmonic resonator. *Opt. Express* **18**, 23633–23645 (2010).

[8] Li, T. E., Subotnik, J. E. & Nitzan, A. Cavity molecular dynamics simulations of liquid water under vibrational ultrastrong coupling. *Proc. Natl. Acad. Sci. U.S.A.* **117**, 18324–18331 (2020).

[9] Yoo, D. et al. Ultrastrong plasmon-phonon coupling via epsilon-near-zero nanocavities. *Nat. Photonics* **15**, 125–130 (2021).

[10] Khitrova, G., Gibbs, H. M., Kira, M., Koch, S. W. & Scherer, A. Vacuum Rabi splitting in semiconductors. *Nat. Phys.* **2**, 81–90 (2006).

[11] Törmä, P. & Barnes, W. L. Strong coupling between surface plasmon polaritons and emitters: a review. *Rep. Prog. Phys.* **78**, 013901 (2015).

[12] Hopfield, J. J. Theory of the contribution of excitons to the complex dielectric constant of crystals. *Phys. Rev.* **112**, 1555 (1958).

[13] Kockum, A. F., Miranowicz, A., De Liberato, S., Savasta, S. & Nori, F. Ultrastrong coupling between light and matter. *Nat. Rev. Phys.* **1**, 19–40 (2019).

[14] De Liberato, S. Light-matter decoupling in the deep strong coupling regime: the breakdown of the Purcell effect. *Phys. Rev. Lett.* **112**, 016401 (2014).

[15] Sauvan, C., Hugonin, J. P., Maksymov, I. S. & Lalanne, P. Theory of the spontaneous optical emission of nanosize photonic and plasmon resonators. *Phys. Rev. Lett.* **110**, 237401 (2013).

[16] Franke, S. et al. Quantization of quasinormal modes for open cavities and plasmonic cavity quantum electrodynamics. *Phys. Rev. Lett.* **122**, 213901 (2019).

[17] Novotny, L. & Hecht, B. *Principles of Nano-Optics* (Cambridge University Press, 2006).

[18] Abujetas, D. R., Feist, J., García-Vidal, F. J., Gómez Rivas, J., & Sánchez-Gil, J. A. Strong coupling between weakly guided semiconductor nanowire modes and an organic dye. *Phys. Rev. B* **99**, 205409 (2019).

[19] Todorov, Y. & Sirtori, C. Intersubband polaritons in the electrical dipole gauge. *Phys. Rev. B* **85**, 045304 (2012).

[20] Zhang, Y., Aizpurua, J. & Esteban, R. Optomechanical collective effects in surface-enhanced Raman scattering from many molecules. *ACS Photonics* **7**, 1676–1688 (2020).

[21] Dicke, R. H. Coherence in spontaneous radiation processes. *Phys. Rev.* **93**, 99 (1954).

[22] Garraway, B. M. The Dicke model in quantum optics: Dicke model revisited. *Phil. Trans. R. Soc. A* **369**, 1137-1155 (2011).

[23] Kaluzny, Y., Goy, P., Gross, M., Raimond, J. M., & Haroche, S. Observation of self-induced Rabi oscillations in two-level atoms excited inside a resonant cavity: the ringing regime of superradiance. *Phys. Rev. Lett.* **51**, 1175 (1983).

[24] Raizen, M. G., Thompson, R. J., Brecha, R. J., Kimble, H. J. & Carmichael, H. J. Normal-mode splitting and linewidth averaging for two-state atoms in an optical cavity. *Phys. Rev. Lett.* **63**, 240 (1989).

[25] Thompson, R. J., Rempe, G. & Kimble, H. J. Observation of normal-mode splitting for an atom in an optical cavity. *Phys. Rev. Lett.* **68**, 1132 (1992).

[26] Wall, F., Mey, O., Schneider, L. M. & Rahimi-Iman, A. Continuously-tunable light-matter coupling in optical microcavities with 2D semiconductors. *Scientific Reports* **10**, 8303 (2020).
